# Intracellular Proteins Targeting with Bi‐Functionalized Magnetic Nanoparticles Following their Endosomal Escape

**DOI:** 10.1002/smll.202410454

**Published:** 2025-02-19

**Authors:** Mélody Perret, Estelle Pineda, Mathilde Le Jeune, Tieu Ngoc Nguyen, Aude Michel, Françoise Illien, Jean‐Michel Siaugue, Christine Ménager, Fabienne Burlina, Emilie Secret

**Affiliations:** ^1^ Sorbonne Université, CNRS Physicochimie des Électrolytes et Nanosystèmes Interfaciaux (PHENIX) 4 place Jussieu Paris 75005 France; ^2^ Sorbonne Université, École Normale Supérieure PSL University CNRS Chimie Physique et Chimie du Vivant (CPCV) 4 place Jussieu Paris 75005 France

**Keywords:** cellular engineering, cytosolic diffusion, endosomal escape, intracellular targeting, magnetic nanoparticles, surface functionalization

## Abstract

The specific targeting of intracellular proteins or organelles by magnetic nanoparticles (MNPs) is a major challenge in nanomedicine, as most MNPs are internalized by cells through endocytosis and remain trapped inside small intracellular vesicles, limiting their ability to reach intracellular components. Furthermore, this phenomenon limits their heating capacity in magnetic hyperthermia, and therefore their potential for cancer treatment. This study presents a strategy based on an original double functionalization of MNPs, with polyhistidine peptides (PHPs) triggering endosomal escape and antibodies targeting specific cytosolic proteins. Negatively charged γ‐Fe_2_O_3_@SiO_2_ MNPs with diameter smaller than 50 nm are functionalized with zwitterionic and thiol groups at their surface. These sulfhydryl groups are used to graft PHPs through a labile link, allowing the peptide to be released from the MNPs’ surface once in the cytosolic reductive environment. This severing avoids any interaction between these peptides and intracellular components, which can hinder MNPs’ intracellular mobility. The second MNPs’ surface functionalization is performed through a non‐labile link with antibodies targeting specific cytosolic proteins, namely HSP27 thermosensitive proteins, for this inaugural proof of concept. Bi‐functionalized MNPs are able to successfully target the intracellular protein of interest, opening the door to promising biomedical applications of MNPs, in cellular engineering and magnetic hyperthermia.

## Graphical abstract

In this work, γ‐Fe_2_O_3_@SiO_2_ core–shell magnetic nanoparticles are functionalized with polyhistidine peptides triggering endosomal escape and antibodies targeting specific cytosolic proteins. Thanks to a smart design of the nanoparticles and of the functionalization strategy, 90% of the internalized nanoparticles are able to target the chosen proteins, opening the door to promising biomedical applications of magnetic nanoparticles.

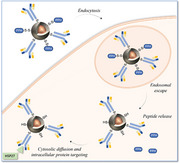

## 1 Introduction

The biomedical field greatly benefits from nanotechnology development. The nanoscale can indeed confer properties that differ from those observed at the macro‐ or microscopic scale. In particular, the size of nano‐objects allows for optimal interaction with biological structures or systems also present at this scale, such as viruses, bacteria, cellular organelles or DNA. That is why a wide variety of nanoparticles are designed for diagnostic, imaging, theragnostic or drug delivery.^[^ ^1^ ^]^ Among them, magnetic nanoparticles (MNPs) have physicochemical properties of great interest for the development of innovative biomedical applications such as imaging, magnetic hyperthermia to selectively destroy cancer cells or to deliver drugs.^[^ ^2^, ^3^, ^4^ ^]^ In addition, these past few years, a new research domain has arisen for MNPs: their use in cellular engineering. In this field, sometimes referred to magnetogenetics, MNPs are used to trigger cellular events, by remotely activating specific components of the cell thanks to MNPs physical properties.^[^ ^5^, ^6^ ^]^ For such applications, MNPs have been mainly designed to activate cellular processes through their interaction with extracellular components. Only a few studies deal with the activation of intracellular components. For example, MNPs have been used to obtain a spatio‐temporal control of intracellular proteins involved in neurite growth,^[^ ^7^, ^8^ ^]^ or to manipulate chromatin inside cell nucleus.^[^ ^9^ ^]^ The rarity of studies involving intracellular interactions between proteins and MNPs is due to MNPs mechanism of internalization when they are incubated with cells. Indeed, small molecules, namely ions, sugars or amino acids, can cross cell membranes through channels or transporters whereas macromolecules or nanoparticles generally enter cells by endocytosis, which involves membrane invaginations to form small vesicles called endosomes that carry the internalized object.^[^ ^10^ ^]^ MNPs remain trapped in endosomes after internalization, which prevents them from freely diffusing inside the cell cytosol to target intracellular components, in addition to losing their heating capacity by magnetic hyperthermia.^[^ ^11^ ^]^ For the time being, applications that require intracellular diffusion of MNPs mainly use MNPs directly micro‐injected inside the cytosol, or techniques such as bead loading or scrape loading.^[^ ^7^, ^8^, ^9^ ^]^ These techniques are stressful for cells, time‐consuming and limited to the treatment of a small number of cells. Therefore, scale up of many applications that involve micro‐injection is unachievable. Accordingly, it is essential to develop MNPs able to overcome this endosomal entrapment.

Endocytic vesicles are subjected to successive transformations during their maturation: their fusion leads to early endosome formation, displaying a luminal pH 6. The content of these sorting endosomes is transferred and accumulated in late endosomes that exhibit a slightly more acidic environment (pH 5). These late endosomes present a light hydrolytic activity and initiate the degradation of internalized objects.^[^ ^12^, ^13^ ^]^ Moreover, this pH decrease induces protonation of amino species having a pKa ≈ 5, which can trigger a proton sponge effect which is described as a massive entry of protons, ions, and water leading to endosome swelling, disruption of their membrane and release of their content in the cytosol.^[^ ^14^, ^15^, ^16^ ^]^ While the exact mechanism remains elusive, endosomal escape of nanoparticles is triggered by the presence of pH‐buffering species such as polyethyleneimine (PEI) or cell‐penetrating peptides (CPP).^[^ ^17^, ^18^, ^19^, ^20^, ^21^ ^]^ Yet, these functionalized MNPs are positively charged at physiologic pH, preventing them from freely diffusing in the cytosol.^[^ ^22^ ^]^ This is why such studies are often focused on the release of proteins, peptides, drugs or nucleic acids, but don't deal with MNPs tracking after their pH‐triggered release.^[^ ^23^, ^24^, ^25^ ^]^ However, MNPs diffusion in the cytosol is required for many applications. Le Jeune *et al.* presented an innovative surface functionalization of MNPs with polyhistidine peptides (PHP), which was shown to promote nanoparticles access to the cytosol.^[^ ^26^ ^]^ Yet, as these peptides were bound to MNPs by a non‐reversible triazole bond, the resulting positively charged MNPs were not able to diffuse freely within the cytosol.

**Figure 1:**
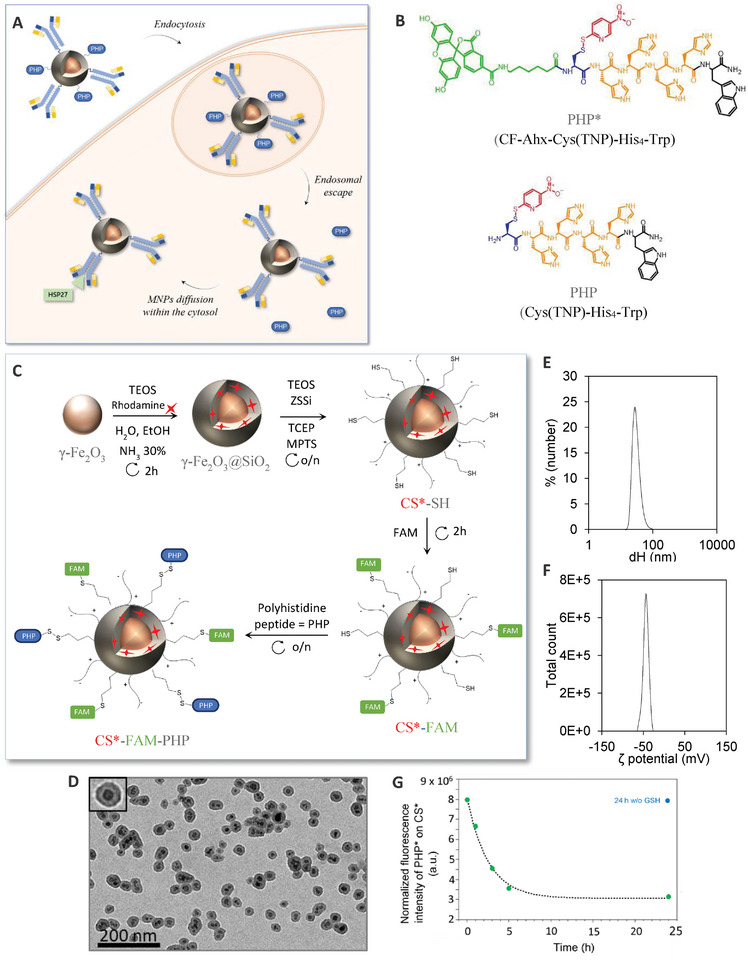
A) Bi‐functionalized magnetic CS nanoparticles internalized by endocytosis, able to reach cell cytosol thanks to PHP and target HSP27 with a specific antibody. B) Chemical structures of the fluorescent polyhistidine peptide CF‐Ahx‐Cys(TNP)‐His4‐Trp (PHP*) and the non‐fluorescent polyhistidine peptide Cys(TNP)‐His4‐Trp (PHP) (black: Trp, orange: His4, blue: Cys, red: TNP, green: CF‐Ahx). C) Synthesis scheme to obtain CS*‐FAM‐PHP. D–F) Characterization of CS*‐FAM‐PHP nanoparticles: (D) transmission electron microscopy image, (E) hydrodynamic diameter determined with DLS and (F) ζ‐potential determined with zetametry. (G) Fluorescence spectroscopy analysis of CF from CS*‐PHP* after GSH reduction (green dots) or after the same incubation time without GSH (blue point), CF fluorescence was normalized with rhodamine fluorescence from CS*.

In this work, we propose a new design for MNPs, capable to escape from endosomes thanks to PHPs, with an evolving surface functionalization in the reducing environment of the cytosol, to ensure efficient diffusion and targeting of specific cytosolic components (**Figure** **1**A). For this proof of concept, we chose as our cytosolic target the heat shock protein HSP27. This temperature‐sensitive protein is of interest for future studies involving magnetic hyperthermia, and involved in cell differentiation mechanisms, making it a prime target for manipulation^[^ ^27^, ^28^ ^]^ in cellular engineering.

To ensure diffusion of MNPs after their endosomal escape, their design needs to respect several criteria, independently of criteria usually used for particles internalization in cells. To promote their diffusion inside the cell cytosol, MNPs should display a diameter smaller than 50 nm, bear a negative charge to limit interactions with intracellular membranes, and present antifouling species onto their surface to limit protein corona formation.^[^ ^22^, ^29^, ^30^ ^]^ For MNPs poly‐functionalization, we exploited thiol chemistry, which provides a wide range of reactions to form reducible (e.g., disulphide) or non‐reducible (e.g., thioether) bonds. To promote MNPs endosomal escape by proton sponge effect, PHPs were grafted at the MNPs surface by a disulphide bond, which can get cleaved in the reducing cytosolic environment.^[^ ^31^ ^]^ This will avoid any side reactions between the peptide's histidine residues and certain intracellular components, such as metallic cations, as well as possible protonation of some of these residues, which could both hinder MNPs diffusion. The targeting molecule, an antibody directed against the HSP27 protein, was instead linked at the MNPs surface via a stable bond (thioether).

To achieve this, spherical magnetic core‐shell (CS) γ‐Fe_2_O_3_@SiO_2_ made of a magnetic iron oxide core and a silica shell to easily functionalize their surface were synthesized. Their colloidal stability was assured by the addition of zwitterionic molecules at their surface, more precisely small sulfobetaines.^[^ ^32^ ^]^ Sulfobetaines have previously been shown to be very efficient at decreasing non‐specific protein adsorption at the surface of nanomaterials,^[^ ^33^, ^34^ ^]^ allowing them to be good candidates to replace poly(ethylene glycol) (PEG) chains that exhibit many drawbacks for biomedical applications, from limiting entry into cells to triggering allergic reactions.^[^ ^35^, ^36^ ^]^ Thiol groups were introduced on CS surface for the subsequent ligation of PHPs through a disulphide bond and the oriented immobilization of anti‐HSP27 antibodies (Ab) through a thioether bond.

Thanks to this original double surface functionalization allowing reversible PHP grafting and stable Ab immobilization, we provide new targeting magnetic nanohybrids that can efficiently reach the cytosol, opening the doors to subcellular environment modulation with magnetic nanoparticles, and could also be engineered for cytosolic delivery applications.

## 2 Results and Discussion

### 2.1 Design of MNPs Able to Access the Cytosol (CS*‐FAM‐PHP)

A five steps synthesis was developed to obtain fluorescent peptides‐coated core‐shell nanoparticles (CS*‐FAM‐PHP) (Figure 1C). First, maghemite nanoparticles, with an average diameter of 11.3 nm (σ = 0.3) were synthesized through an alkaline co‐precipitation of iron salts, followed by a size sorting procedure in order to decrease their polydispersity^[^ ^37^ ^]^ (Figure S1A,B, Supporting Information). Then, the silica shell was formed through a Stöber process, hence a condensation of tetraethylorthosilicates (TEOS) in alkaline conditions. The surface of the core‐shell MNPs synthesized was then functionalized by silanization. To prevent the formation of a protein corona and promote CS cellular uptake, the surface was silanized using an organofunctional alkoxysilane containing a sulfobetaine group (3‐{[dimethyl(3‐trimethoxysilyl)propyl]ammonio}‐propane‐1‐sulfonate, called ZSSi in this publication). Thiol groups were introduced onto CS surface with an average number of 1250 sulfhydryl functions per composite by grafting (3‐mercaptopropyl)triethoxysilane (MPTS) alongside ZSSi (See material and methods section). MNPs can be made fluorescent by adding a silanized fluorophore, such as rhodamine‐B (CS*‐SH, each fluorescent specie of this manuscript being labeled with an *) during the formation of the silica layer.^[^ ^26^, ^38^ ^]^ These CS*‐SH were characterized with transmission electron microscopy (TEM), dynamic light scattering (DLS) and ζ‐potential measurements (Figure S1, Supporting Information). Spherical MNPs were made of an average of 2 magnetic cores per silica shell. CS*‐SH displayed a hydrodynamic diameter of 40 nm, smaller than 50 nm which is required for intracellular diffusion, and a negative surface charge (−47 mV) that limits interactions between nanoparticles and cell membranes that could block their diffusion.^[^ ^22^ ^]^

In order to track MNPs inside cells with fluorescence‐based technics (microscopy, flow cytometry), it is necessary to be able to quench any fluorescence that could come from the non‐internalized membrane‐bound MNPs. Fluorophores inside the silica shell are not accessible to quenching molecules, hence the necessity to also graft a fluorescent probe onto the CS surface. So, a fluorescein‐maleimide (FAM) was grafted on half of the thiol groups present at the surface of CS*‐SH MNPs via a stable thioether bond (CS*‐FAM). Fluorescein was chosen here because its fluorescence can be quenched using trypan blue. Importantly, trypan blue is not internalized inside cells, providing a method to exclusively quench fluorescein fluorescence of extracellular membrane‐bound CS.^[^ ^26^ ^]^ These CS*‐FAM nanoparticles still displayed a hydrodynamic diameter of 40 nm with a negatively charged surface (Table S1, Supporting Information).

In order to react with these MNPs to form a disulphide bond, two polyhistidine peptides, one fluorescent (PHP*) and the other non‐fluorescent (PHP), were synthesized by Fmoc solid phase peptide synthesis (Figure 1B). In addition to four histidine residues to trigger endosomal membrane disruption by a proton sponge effect, a cysteine was added. A tryptophan was also integrated into the peptide to simplify peptide purification and handling. For the fluorescent peptide, a fluorescein was attached at the N‐terminal extremity, spaced from histidines with a carbon chain (aminohexanoic acid, Ahx). Peptide final deprotection and cleavage from the resin was performed by treatment with trifluoroacetic acid in presence of dithiobis(5‐nitropyridine) (DTNP). This directly provided the peptide activated on the thiol group of its cysteine residue by a nitropyridine sulfenyl (TNP) moiety, to promote the subsequent disulphide conjugation of the peptide to the free sulfhydryl functions of CS*‐SH or CS*‐FAM. PHP and PHP* were purified by reverse phase high performance liquid chromatography (HPLC) (Figure S2A,B, Supporting Information). Peptide characterization was performed by matrix assisted laser desorption ionization – time of flight mass spectrometry (MALDI‐TOF MS) analysis in positive linear mode (Figure S2C,D, Supporting Information). The m/z values for both PHP* and PHP [MH]+ protonated species were observed at 1481.3 and 1009.9 respectively as expected. Peaks resulting from fragmentations during MS analysis were also observed (Table S2, Supporting Information).

To verify mono‐functionalization of MNPs with peptides through a cleavable linker, CS*‐SH nanoparticles were first functionalized by a disulphide bridge with fluorescent PHP*, without FAM (Figure S3, Supporting Information). Rhodamine incorporation inside the silica shell allowed for PHP* grafting yield estimation. Noteworthy, quenching of PHP* fluorescence was observed when the peptide was attached on MNP surface, complicating the estimation of the grafting yield. To overcome this problem, samples of CS*‐PHP* at different concentrations were treated with a reducing agent (tris(2‐carboxyethyl)phosphine hydrochloride, TCEP) and the fluorescence of the released PHP* was measured. The fluorescence intensity from the free peptide was then found to be proportional to CS iron concentration (Figure S3F, Supporting Information) and the obtained determination coefficient confirmed the total reduction of PHP* from CS surface. By referring to a PHP* fluorescence standard curve (Figure S3G, Supporting Information), an average peptide grafting yield of 14,4 ± 1,5% was calculated, which corresponded to an average number of 180 PHP* per particle.

To ensure that intracellular glutathione (GSH) would be able to reduce the disulphide bonds linking the PHP* to CS, an in vitro cleavage assay was also conducted. To mimic cells conditions, GSH was used at its estimated concentration in the cytosol, i.e., 10 mM^[^ ^31^ ^]^ and mixed with CS*‐PHP* for 0, 1, 3, 5, or 24 h. The cleaved PHP* was then eliminated by ultra‐filtration and fluorescence of the remaining peptide on CS* surface was measured (Figure 1G). As expected, PHP* fluorescence intensity after 5 h of reaction with GSH had already intensely decreased, and was lowering down until 24 h of reduction. In addition to proving that GSH was able to release PHP* from CS surface, these results also confirmed that PHP* was covalently grafted onto CS and not only adsorbed at their surface since cleaved PHP* quantity decreased with the time of treatment with GSH.

In order to obtain CS*‐FAM‐PHP nanoparticles to evaluate their ability to reach the cytosol, this double functionalization of MNPs with fluorescent probes and PHPs was verified by fluorescence spectroscopy and confocal microscopy experiments on live cells. It should be note that all colocalization analyses performed during this study were conducted using Manders’ coefficients along with statistical significance verification (see Supporting Information).^[^ ^39^, ^40^, ^41^ ^]^ First, grafting of the fluorescein FAM was confirmed by its colocalization with rhodamine inside CS* (Figures S4D,E and S5A—F, Supporting Information). Once the grafting of this fluorescent probe through a thioether bond was confirmed, this functionalization was combined with peptides immobilization. To do so, the fluorescein maleimide was temporary replaced with a rhodamine maleimide (ROX) to avoid overlap of fluorescence with the green fluorescent PHP*. CS‐ROX‐PHP* synthesis was confirmed by colocalization between ROX and PHP* (M1 = 0.763 and M2 = 0.804) (Figure S5G,I—K, Supporting Information). Eventually, this grafting strategy was used to obtain CS*‐FAM‐PHP nanoparticles: non‐fluorescent PHP was grafted onto CS surface via a disulphide bond along with fluorescein‐maleimide (FAM) via a thioether bond, giving CS*‐FAM‐PHP nanoparticles. A TEM image of CS*‐FAM‐PHP is presented (Figure 1D). CS*‐FAM‐PHP still exhibited a hydrodynamic diameter smaller than 50 nm and a negative surface charge (‐ 44 mV) (Figure 1E,F; Table S1, Supporting Information).

### 2.2 Evaluation of MNPs Cytosolic Diffusion

Before conducting cells studies, CS colloidal stability in cell culture media (DMEM/F12) was monitored by DLS (Figure S6A, Supporting Information). Particles colloidal stability was at first not affected by their transfer in cell medium. Aggregation of particles functionalized with FAM occurred after at least 3 h of incubation time, while particles functionalized with PHP* only start aggregating after 4 h of incubation, which would leave enough time for CS to be internalized in cells.^[^ ^26^ ^]^ Cytotoxicity evaluation was conducted with a lactate dehydrogenase (LDH) assay (Figure S6B, Supporting Information). SH‐SY5Y cells were incubated with CS*‐SH and CS*‐PHP* particles at different iron concentrations (0.5, 1.0, 3.0, and 5.0 mm) in culture medium for 4 h. Fresh supplemented culture medium was then added and cell death was measured after a total incubation time of 48 h. A very small cytotoxicity was measured for CS*‐SH particles, below 15%, while no cytotoxicity was observed for CS*‐PHP*. CS*‐FAM‐PHP ability to escape endosomes and reach the cytosol was evaluated by confocal microscopy. It was first necessary to determine the best duration of incubation of cells with MNPs in order to observe endosomal escape (Figure S7, Supporting Information). Depending on the cell type, the required time for endocytosis to occur can indeed vary, as well as the time for endosomes maturation. For SH‐SY5Y cells, confocal microscopy images were taken 24 h after incubation of particles: cells were incubated with MNPs for 4 h, washed and incubated with culture medium without MNPs for 20 h before imaging. This procedure enabled to limit particles deposition around cells that could interfere during fluorescence image acquisition. Importantly, it gave enough time for the internalized MNPs to escape from endosomes and reach the cytosol leading to PHP* release from the MNPs surface.

**Figure 2:**
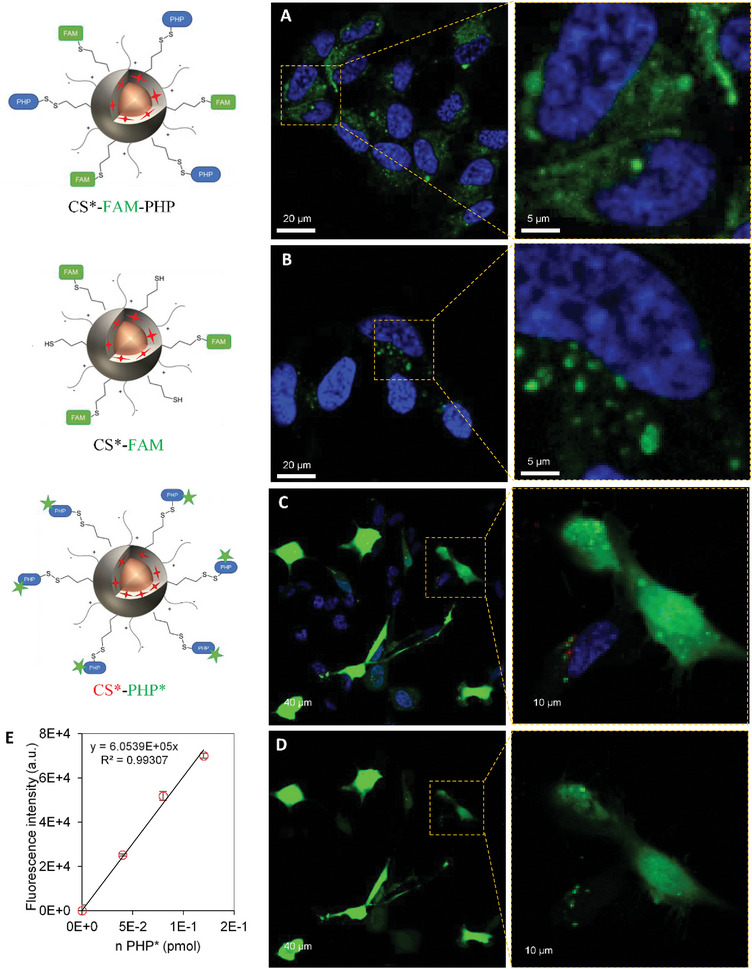
A–D) Confocal microscopy images on live SH‐SY5Y cells, incubated with MNPs at [Fe] = 1 mm. Cells were incubated for 4 h with nanoparticles, washed, and observed 20 h later. Cell nuclei are in blue (Hoechst dye 33342). A,B) Green fluorescence from FAM on CS surface, cells were treated with trypan blue: A) CS*‐FAM‐PHP and B) CS*‐FAM. (C‐D) CS*‐PHP* green fluorescence from CF on PHP*: C) Merged channels with red fluorescence from CS* and green fluorescence from PHP*, D) same cells treated with trypan blue. E) PHP* fluorescence standard curve measured in the presence of the lysate of 1 million cells, as a function of the number of moles of PHP* added.

SH‐SY5Y cells were incubated with CS*‐FAM‐PHP particles and imaged by confocal microscopy (**Figure** **2**A). Trypan blue was used to quench FAM green fluorescence outside cells in order to only observe internalized nanoparticles. The green fluorescence corresponding to CS appeared significantly diffuse in the cytosol. The distribution of MNPs’ fluorescence in the whole cytosol demonstrated that PHP functionalization onto CS surface triggers endosomal escape of nanoparticles, then able to reach the cytosol. Some dots of green fluorescence corresponding to CS trapped in vesicles were still observed, suggesting that not the totality of CS escaped endosomes, or that they were picked up by cell defense mechanisms after their escape from endosomes. On the contrary, when cells were incubated with CS*‐FAM without PHP, green fluorescence from FAM appeared punctuated, suggesting that MNPs remained trapped in endosomes (Figure 2B). Therefore, the presence of polyhistidines peptides onto CS surface is mandatory for pH‐triggered release of MNPs in the cytosol. A sample of PHP* fluorescent peptide (not linked to CS) was also incubated with cells and the resulting confocal images showed only few green fluorescence dots from the peptide (Figure S8A, Supporting Information). This showed that this small peptide by itself is poorly internalized and that it requires nanoparticles to trigger endocytosis. Noteworthy, an increase in peptides number onto CSs surface did not result in a stronger fluorescence within the cytosol (See Figure S8, Supporting Information).

Peptide release from MNPs surface after triggering their endosomal escape was assessed by confocal microscopy on CS*‐PHP* samples and colocalization analysis. The fluorescence signals from CS* in red and from PHP* in green were not colocalized when merged (Figure 2C) as shown by the corresponding Manders’ coefficients (M1 = 0.942 and M2 = 0.017) (Figure S5H, Supporting Information). The value for M2 is significantly inferior from M1: red signal (CS*) was always associated with green signal (PHP*), whereas green signal could be found alone (free PHP*). These results confirmed that CS could escape endosomes, most probably by proton sponge effect, and PHPs* were released from CS surface after 24 h of incubation thanks to the cell reducing environment. The green fluorescence from PHP* was distributed within the whole cell, the peptide was even able to reach cell nuclei (Figure 2C), which is likely for the free peptide but would not happen if it was still attached onto NP surface. A difference in fluorescence emission was observed after adding trypan blue. This phenomenon likely results from extinction of the fluorescence of peptides bound to the surface of cells.^[^ ^42^ ^]^ Images obtained with and without trypan blue treatment are not strongly different, which might reflect a self‐quenching of the fluorescein peptide at the cell membrane, as previously observed for cell‐penetrating peptides (CPP)^[^ ^43^, ^44^ ^]^ (Figure 2C,D respectively).

Since this system could be valuable for drug delivery, an estimation of the amount of PHP* internalized in cells was performed. To do so, cells were incubated with CS‐PHP* at a 1 mm iron concentration, according with cytotoxicity evaluations (Figure S6B, Supporting Information). To directly measure the amount of internalized PHP* when cells were incubated with CS*‐PHP*, the protocol developed by Illien *et al.* to quantify the internalization of cell‐penetrating peptides (CPPs) by fluorescence spectroscopy was adapted here to the functionalized CS.^[^ ^44^ ^]^ When PHP* are grafted on CS surface, their fluorescence is quenched which prevents their quantification. However, when CS are internalized in cells and escape from endosomes, PHP* get released from CS surface thanks to the cytosolic reducing environment, which induces fluorescence dequenching and also allows PHP* separation from CS after cells lysis. Along with a pre‐treatment of cells with TCEP and trypsin/EDTA (or pronase) to remove all PHP* potentially bound to the cell surface,^[^ ^45^ ^]^ it was then possible to eliminate cellular remains as well as CS and recover only PHP* delivered inside cells to measure fluorescence without any quenching phenomenon. The efficacy of TCEP and trypsin/EDTA (or pronase) treatment to remove all membrane‐bound species was first verified with flow cytometry (see supplementary, Figure S9, Supporting Information). The quantification by fluorometry of peptides internalized inside cells was then performed referring to a standard curve of PHP* fluorescence as a function of the number of moles of PHP* added to cell lysates, for which cells auto‐fluorescence was deducted, as for the internalized samples (Figure 2E). Using this method, an average of 2.6 ± 0.8 pmol of PHP* internalized in one million cells was measured. This corresponds to an intracellular peptide concentration of ≈1.3 µm (using an estimated volume for a SH‐SY5Y cell of 2 pL). By referring to the grafting yield of PHP* onto CS and the amount of iron per CS, this value corresponds to an average of 10 000 MNPs in one cell. This experiment thus allowed a precise quantification of internalized species and importantly it showed that this system can deliver compounds in the cytosol in the µmolar range concentration. In addition, fluorescence from a control sample without treatment with TCEP, trypsin and EDTA was measured, giving access to the total amount of peptide associated with cells, including the membrane‐bound and internalized species. This was compared to the result obtained with the treated samples. From this comparison, it was calculated that 68.9 ± 0.3% of peptides associated with cells are membrane‐bound, while 31.1% of peptides, hence of MNPs, are actually internalized species.

The MNPs synthesized here display a size and surface charge suitable for intracytosolic diffusion, as well as an innovative labile functionalization that relies on the cellular machinery. Altogether, these results show that it is possible to obtain magnetic nanoparticles able to diffuse in the cytosol. The surface of these particles was then tuned to target a specific intracellular protein after endosomal escape of MNPs.

### 2.3 Bi‐Functionalization of CSs with Polyhistidine Peptides and Antibodies

To specifically target the intracellular thermosensitive protein HSP27, a double surface functionalization of MNPs was conducted, with PHP to reach the cytosol and with an antibody as targeting species (CS*‐PHP‐Ab*). A fluorescent anti‐HSP27 antibody (Ab*) was used here as a proof of concept. We chose to make an oriented immobilization of the Ab in order to keep the antigen binding sites available, directed outward from the particle, thus maintaining the antibody recognition of its antigen (Figure S10A, Supporting Information). Immunoglobulins present a wide variety of reactive functional groups such as amino groups or carboxylic acids. However, these groups are distributed all over the antibody, preventing their use for an oriented grafting. Several techniques are presented in the literature to make antibody‐enzyme conjugates and often require antibody partial reduction.^[^ ^46^, ^47^ ^]^ Jeong *et al.* ^[^ ^48^ ^]^ adapted a well‐known strategy for IgG oriented immobilization onto nanoparticles. It consisted in the partial reduction of Ab to obtain free sulfhydryl groups on their heavy chains, that will later react with NP surface (Figure S10B, Supporting Information). Indeed, disulphide bonds are connecting the heavy chains together, but also the light ones. Reduction of the latter would lead to a loss of the antigen recognition site. Therefore, it is necessary to carry out a partial and controlled reduction, mostly directed toward the disulphide bonds connecting the heavy chains, more accessible to the solvent, sparing the ones between the light chains to obtain mainly bio‐active forms of reduced Ab.^[^ ^46^ ^]^ GSH and other reducing systems that can be found in the cytosol are unable to reduce these buried disulphide bonds: the 3D conformation of antibodies prevents them from reaching their functions. Thus, such reduction can be achieved in solution but will not occur once in cells. Partial reduction of the Ab* was conducted with TCEP and re‐oxidation of Ab* moieties was prevented using EDTA.^[^ ^47^ ^]^ Different reducing agent ratios were tested and the best conditions were determined with SDS‐PAGE analysis (sodium dodecyl sulfate – polyacrylamide gel electrophoresis). For the Ab* used in this study, the band corresponding to the separation of the two heavy chains started appearing with 10 eq of TCEP. However, the 80 and 45 kDa bands, corresponding to biologically inactive fragments, were also visible (Figure S10C, Supporting Information). Higher concentrations of TCEP lead to higher amounts of inactive fragments. Higher molecular weight signals corresponded to the full Ab. This bioactive form could present free thiol groups even without complete separation of both heavy chains and could therefore be suitable for an Ab oriented immobilization. 10 eq of TCEP appeared to be the best amount of reducing agent in order to reduce the anti‐HSP27 Ab* to half Ab, which would lead to a decrease of its steric hindrance once attached to the MNPs, while maintaining the antigen binding site's integrity.

Unlike the polyhistidine peptide, the antibody must be grafted onto CS surface with a non‐labile link, so that it stays on the CS surface after internalization, and can target its intracellular antigen. The use of maleimide groups meets these criteria, since when reacting with thiol functions it gives a stable thioether link. Therefore, to connect sulfhydryl groups from reduced antibodies to thiol‐coated CS, a maleimide homo‐bis‐linker was used. 1,4‐bis(maleimido)butane (BMB) has two maleimide groups, one that can react with thiols on CS and the second one with antibodies. The main risk while modifying thiol groups at the surface of MNPs with BMB is the formation of intra or inter‐particles thioether bonds that could lead to CS destabilisation and aggregation. This would also decrease the number of maleimide groups available at NP surface and limit the immobilization of Ab. An excess of BMB per thiol groups was therefore introduced to avoid these secondary reactions, keeping the MNPs stable and allowing their further functionalization (Figure S10D, Supporting Information).

**Figure 3:**
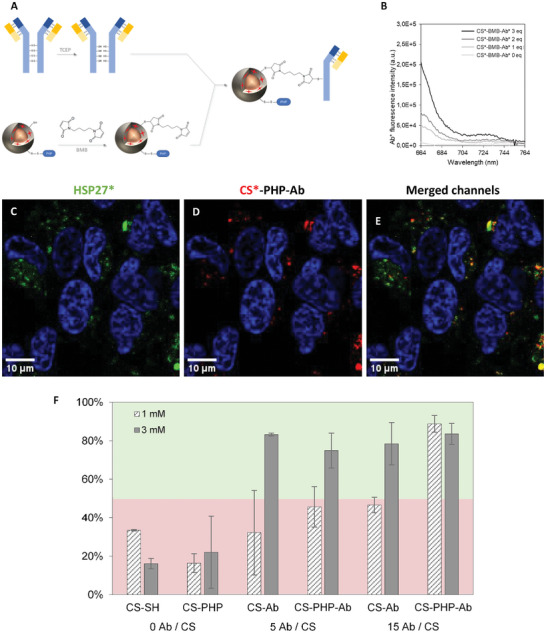
A) Double surface functionalization of MNPs with PHP and Ab reaction scheme. B–E) Evaluation of MNPs targeting capacity toward HSP27. B–D) Confocal microscopy images on live SH‐SY5Y cells, incubated 4 h with CS*‐PHP‐Ab functionalized with 3 Ab/CS at [Fe] = 3 mm and observed 24 h after incubation. B) green fluorescence from GFP on HSP27*, C) red fluorescence from rhodamine inside CS and (D) merged channels. Cell nuclei in blue (Hoechst dye 33342). E) M1 coefficients determined by colocalization analysis from confocal microscopy images on cells incubated with different MNPs (functionalized with 0, 1 or 3 Ab/CS), at different iron concentrations (1 mm or 3 mm). The exact M1 and M2 values are shown in Table S3 (Supporting Information).

Our double functionalization strategy relied in first grafting the PHP onto half of the thiol functions present on CS surface, then reacting the other half with BMB molecules, to finally conjugate the reduced Ab* (**Figure** **3**A). This sequence of grafting was chosen for several reasons: to avoid free maleimide functions reacting with the other half of thiol functions of CS surface that are required for PHP grafting, to avoid steric hindrance from the antibodies that could block the PHP from reacting onto CS and also to prevent cross reactions between the peptide and the Ab sulfhydryl groups. In order to verify the double functionalization with fluorescence spectroscopy, each species was labelled with different fluorescent probes: CS silica shell was loaded with rhodamine (CS*), the fluorescein‐labelled peptide was used (PHP*) and the Ab presented an allophycocyanin (APC) fluorophore (Ab*). All of these species emitted a fluorescence signal after CS*‐PHP*‐Ab* synthesis (Figure S11C—E, Supporting Information). APC signal remained low because it was present in smaller quantity compared to both other fluorophores, but its emission intensity was increased along with the amount of Ab* introduced onto CS surface showing its successful immobilization on particles (Figure S11A,B, Supporting Information). To check that Ab grafting remained stable as required in the intracellular environment, cells were incubated with CS*‐PHP*‐Ab* and observed with confocal microscopy 24 h after incubation (Figure S11F—I, Supporting Information). The obtained images confirmed the presence of Ab* at the surface of CS, whereas the PHP* appeared uncolocalized with the MNPs as expected after endosomal escape. After confirmation of this double functionalization and cytotoxicity evaluation (Figure S6B, Supporting Information), the following experiments were conducted with a non‐fluorescent peptide (CS*‐PHP‐Ab*).

### 2.4 Intracellular Protein Targeting with MNPs

MNPs ability to target intracellular HSP27s was evaluated by colocalization analysis on confocal microscopy images (Figure 3E; Table S3; Figures S13–S15, Supporting Information) between fluorescent nanoparticles (rhodamine in the silica shell) and fluorescent HSP27*, obtained after transfection of SH‐SY5Y cells with a recombinant plasmid to express fluorescent HSP27‐GFP (Figure S12, Supporting Information).

Two different amounts of MNPs in cells were evaluated: cells were first incubated with MNPs using a 1 mm iron concentration, then a threefold higher amount of MNPs was tested (3 mM iron concentration of incubation), the number of Ab grafted on CS surface was also varied. Percentages of MNPs that targeted HSP27* are shown in Figure 3E. They correspond to Manders’ coefficient M1 (ratio of the red CS* fluorescence overlapping the green HSP27 fluorescence).

#### 2.4.1 Targeting Capacity of MNPs When Using a 1 mm Iron Concentration

First, control samples without antibodies nor PHP (CS*‐SH) were imaged in cells, as well as particles able to reach the cytosol but that don't possess a targeting agent (CS*‐PHP) (Figure S13A,B respectively, Supporting Information). In both cases, as expected, MNPs were not able to target HSP27s*. M1 coefficients were indeed smaller than 35% (Figure 3E) and statistical significance analysis showed that the detected colocalization was random.

To assess MNPs behavior in cells without PHPs, cells were first incubated with CS*‐Ab* functionalized with 1 Ab per CS (Figure S14A, Supporting Information). CS red fluorescence appeared punctuated and no significant colocalization was observed between CS* and HSP27* on these images (M1 = 32.3 ± 22.0%), in agreement with an endosomal localization of these particles without PHP (Figure 3F). To enhance the MNPs targeting capacity (with M1 > 50%), the number of Ab* grafted onto CS*‐Ab* surface was increased from 1 to 3 Ab per CS. Surprisingly, the incubation of these MNPs led to a better colocalization between MNPs and HSP27* (M1 = 46.7 ± 4.0%) (Figures 3F and S14C, Supporting Information). Contrary to MNPs without Ab, the detected colocalization was here statistically significant and did not result from random colocalization. These first results suggest that a portion of CS* succeeded in targeting HSP27. Theoretically, despite the increase in Ab grafting, no colocalization was meant to occur for CS*‐Ab* since these particles have no PHP on their surface to trigger endosomal escape. This result could unveil antibodies ability to trigger endosomal escape of particles in the same way as the PHP.

When incubating cells with bi‐functionalized particles (CS*‐PHP‐Ab*), colocalization seemed to improve even more (Figure 3E). With 1 Ab/CS, the M1 coefficient value reached 50%, indicating that a fraction of incubated CS was able to target the protein of interest (Figure S14B, Supporting Information). Red fluorescence of particles again appeared punctuated because part of CS is concentrated around HSP27s, and part of them might still be blocked in endosomes. Gratifyingly, increasing the number of grafted Ab to 3 Ab/CS resulted in a much better colocalization between particles and HSP27s with a M1 coefficient reaching this time 89% (Figures 3E and S14D, Supporting Information). These conditions of MNP surface bi‐functionalization therefore featured a strong targeting capacity of the protein of interest.

Eventually, the presence of Ab onto MNPs surface was mandatory to target HSP27. The increase in the number of Ab on CS led to better targeting efficiency, as they were statistically more likely to associate with a protein, despite the conformational constraints generated by their covalent grafting on the particle surface.

#### 2.4.2 Targeting Capacity of MNPs When Using a 3 mm Iron Concentration

To reach a higher number of particles in the cell and achieve more targeting of HSP27, cells were incubated with a higher amount of MNPs.

To begin with, surface functionalization of CSs was maintained at 1 Ab/CS. Every type of MNPs presented so far were tested again for a cell incubation with three times higher iron concentration (3 mm). Control CS without antibodies, CS*‐SH and CS*‐PHP (Figure S13C,D respectively, Supporting Information) maintained low Manders’ coefficients similar to those obtained for a 1 mm iron concentration (Figure 3E).

Already, an effect of the increase of MNPs in cells was observed for particles without peptide, again with 1 Ab/CS (Figure S15A, Supporting Information). Indeed, 83% of internalized particles were able to target proteins compared to 32% with a 1 mm incubation (Figure 3E). This increase of M1 coefficient was even more pronounced than when increasing the number of antibodies grafted onto particles surface. A threefold higher iron concentration appeared more effective in improving targeting than tripling the quantity of antibodies grafted on the CSs since more MNPs fluorescence can be detected in cells. CS functionalized with 3 Ab/CS, still without PHP, displayed a high targeting efficiency with a M1 coefficient near 80% (Figures 3E and S15C, Supporting Information). The increase in iron concentration, therefore in Ab concentration in endosomes after particles internalization, resulted in a drastic hike of interactions between MNPs and HSP27s. Here again, the antibody seemed to trigger endosomal escape of particles, then able to reach the cytosol and target HSP27, even without PHPs.

When CS presented peptides on their surface, results were quite similar. An increase in the iron concentration for cells incubation with CS*‐PHP‐Ab* didn't show significative changes regarding HSP27 targeting (Figures 3E and S15B,D, Supporting Information). Nevertheless, these bi‐functionalized MNPs, incubated at 1 mm or 3 mm, always displayed the highest Manders’ coefficients. Indeed, for CS*‐PHP‐Ab* functionalized with 3 Ab/CS, 83,6 ± 5,4% of MNPs were able to efficiently target the protein of interest inside the cytosol (Figure 3B—E).

Colocalization quantification in cells between MNPs and HSP27s enabled the evaluation of particles targeting ability toward these intracellular proteins. Once again, these results confirmed the capacity of such MNPs to reach the cytosol thanks to a surface functionalization with polyhistidine peptides. When MNPs exhibited a bi‐functionalization with PHPs and anti‐HSP27 antibodies, particles were able to target this intracellular protein and this capacity was increased along with the number of Ab grafted onto CSs surface. Surprisingly, under certain conditions, the Manders coefficients values evoked interactions between Ab‐functionalized CSs and HSP27s even when no PHP was present on the CSs. M1 values for bi‐functionalized samples were always ≈90%, when coefficients corresponding to particles with Ab and without PHP kept increasing. These results suggest that some amino‐acids present in the Ab could be protonated in late endosomes, and would allow, like PHPs, to free particles in the cytosol. Another mechanism of endosomal membrane destabilization may also be involved. This somewhat unexpected result may have important implications in the delivery field. It suggests that functionalization of delivery systems with targeting antibodies can in some cases promote endosomal escape. If it is required to maintain a low number of MNPs in the cell, it is possible to work with a smaller particle concentration within cells, in which case the presence of polyhistidine peptides is mandatory to obtain freely diffusing particles. Similarly, the overall cost of such objects can be significantly decreased if the peptide is used and the number of antibodies is lowered. The use of polyhistidine peptides would also be necessary if combined to other types of targeting agents on MNPs (targeting peptides, aptamers, HaloTag ligand, small molecules…).

## 3 Conclusion

Through the combination of a labile surface functionalization of MNPs with molecules that can induce a proton sponge effect (PHPs) and a stable functionalization with targeting species (Ab), the proposed particles are able to bypass the natural vesicular traffic within cells. This enables them to reach the cytosol where they can regain their mobility and are then able to target an intracellular protein of interest. Without microinjecting particles directly inside the cytosol, the only examples of intracellular targeting with MNPs are, to our knowledge, mitochondria targeting, often using a triphenylphosphine functionalization that can act as a proton sponge species and a mitochondria targeting agent.^[^ ^49^, ^50^, ^51^ ^]^ The system presented here is more versatile, enabling for the first time the targeting of any intracellular protein or organelle with magnetic nanoparticles after a standard cellular incubation, just by changing the targeting agent. Eventually, we provide new magnetic nano‐objects that enable scale up of many applications of MNPs in the biomedicine field. For example, in case of cancer treatment, the labile surface functionalization of such particles could be used for drug or SiRNA delivery and cytosolic release, paired with targeting of specific proteins or compartments such as mitochondria. In fact, the presence of a silica shell and thiol functions around iron oxide NPs provide an anchor point for many molecules that enables an easy modulation of MNPs surface. All these concepts can also be coupled with magnetic targeting, magnetic hyperthermia or imaging, thanks to properties that are exhibited by iron oxide nanoparticles. Here, HSP27s were targeted as a proof of concept since such proteins are sensitive to changes in temperature that can be obtained by magnetic hyperthermia. Their modulation thanks to the effect of MNPs could lead to new cellular engineering pathways.

## 4 Experimental Section

### Materials

Standard Fmoc amino acids were acquired from Iris Biotech (Germany). Rink amide AM resin, N,N,N,N‐tetramethyl‐O‐(1H‐benzotriazol‐1‐yl)uroniumhexafluorophosphate (HBTU), N‐diisopropylethylamine (DIEA), Fmoc aminohexanoic acid (Fmoc‐Ɛ‐Ahx‐OH), N,N‐diisopropylcarbodiimide (DIC), 1‐hydroxybenzotriazole hydrate (HOBt), 2,2′‐dithiobis(5‐nitropyridine) (DTNP), sodium citrate dihydrate, tetraethyl orthosilicate (TEOS), tris(2‐carboxyethyl)phosphine hydrochloride (TCEP), sodium phosphate, sodium chloride, tris(hydroxymethyl)aminomethane, glycerol, dimethylsulfoxide (DMSO), N‐(2‐hydroxyethyl)piperazine‐N’‐(2‐ethanesulfonic acid) (HEPES), ethylenediaminetetraacetic acid (EDTA), rhodamine B isothiocyanate (Rhod), (3‐aminopropyl)triethoxysilane (APTS), 5,5‐dithio‐bis‐(2‐nitrobenzoic acid) (DTNB), 3‐(N‐morpholino)propanesulfonic acid (MOPS), Dulbecco's phosphate‐buffered saline (DPBS), Hanks’ balanced salt solution (HBSS) and ultrafiltration columns Amicon Ultra – 100 kDa, were purchased from Sigma‐Aldrich (France). Piperidine (peptide synthesis grade), dimethylformamide (DMF) (peptide synthesis grade), dichloromethane (DCM) (analysis grade), trifluoroacetic acid (TFA) (optical spectroscopy grade), acetonitrile (ACN) (high‐performance liquid chromatography, HPLC grade), ammonia (22.5% and 30%) and diethyl ether were obtained from Carlo Erba (France). Iron(II) chloride tetrahydrate, iron(III) chloride hexahydrate, iron(III) nitrate nonahydrate, hydrochloric acid fuming (37%), nitric acid (52.5%), acrylamide/bisacrylamide, sodium dodecyl sulfate (SDS), tetramethylethylenediamine and bromophenol blue, acetone and ethanol absolute were purchased from VWR (France). (3‐mercaptopropyl)triethoxysilane (MPTS) was purchased from ABCR (Germany). 3‐{[dimethyl(3‐trimethoxysilyl)propyl]ammonio}propane‐1‐sulfonate (ZSSi) was obtained from Gelest (USA). 6‐FAM‐maleimide (FAM) and 5‐ROX‐maleimide (ROX) were acquired from Lumiprobe (France). Dulbecco's modified eagle medium/nutrient mixture F‐12 (DMEM/F12), heat inactivated fetal bovine serum (HI FBS), trypsin/ethylenediaminetetraacetic acid (Trypsin/EDTA) and fetal bovine serum (FBS) were purchased from PAN‐Biotech (Germany). Penicillin/streptomycin (pen/strep) was obtained from Dutscher (Germany). Kanamycin was bought from Gold Biotechnology (USA). Trypan blue was purchased from Corning (France). Fluorescent anti‐HSP27 monoclonal antibody APC (#MA545205, Lot YF3937111), 1,4‐bismaleimidobutane (BMB), PageRuler Plus Prestained Protein Ladder, ammonium persulfate, GeneJET Plasmid Miniprep Kit, Gibco Opti‐MEM reduced serum medium (Opti‐MEM), Luria Broth (LB), Nucblue Live Cell Stain Readyprobes reagent (Hœchst 33342 dye), CyQUANT lactate dehydrogenase (LDH) cytotoxicity assay kit and Lipofectamine 3000 reagent were bought from Thermo Fisher Scientific (USA). The equipment to pour, cast and migrate SDS‐PAGE gels and the ChemiDoc Imaging system were bought from Bio‐Rad (USA). Plasmid pEGFP‐hsp27 wt FL was a gift from Andrea Doseff (Addgene plasmid #17444, http://n2t.net/addgene:17444; RRID:Addgene_17444).^[^ ^52^ ^]^

### Peptide Synthesis

Polyhistidine peptides (PHP: H‐Cys(TNP)‐His‐His‐His‐His‐Trp‐NH_2_ and PHP*: Fluorescein‐Ahx‐Cys(TNP)‐His‐His‐His‐His‐Trp‐NH_2_) were synthesized manually by standard Fmoc solid‐phase peptide synthesis on rink amide AM resin (0.35 mmol g^−1^ resin, 0.1 mmol scale) using Fmoc‐protected amino acids (5 eq.) and Fmoc‐protected aminohexanoic acid (Ahx) activated with HBTU (4.5 eq.) and DIEA (10 eq.) in DMF (3 min activation followed by 30 min coupling at RT). The Fmoc protecting group was eliminated at each cycle with a 20% v/v piperidine solution in DMF (3 + 7 min). 5(6)‐carboxyfluorescein (5 eq.) was coupled on the N‐terminus of PHP* with DIC (5 eq.) and HOBt (10 eq.) in DMF (activation for 10 min followed by 15 h of coupling at RT), the resin was then washed with a 20% v/v piperidine solution in DMF. At the end of synthesis, both peptide‐resins were washed with DMF, DCM and methanol and vacuum‐dried for 2 h. Final deprotection and cleavage of both peptides from the resins were performed by 2 h treatment with a solution of 97.5% of TFA, 2.5% of TIS. DTNP (3 eq.) was added to this solution to activate the cysteine residue with a NPyS group. TFA was sparged under argon. Then, peptides were precipitated in cold diethyl ether for 48 h at ‐20 °C, the samples were centrifuged to pellet the peptides and the ether was removed. The pellets were dissolved in 0.1% TFA in H_2_O and lyophilized.

By‐products were removed with reverse‐phase HPLC (RP‐C18 column, 5 µm, 250 × 16 mm, Macherey Nagel) with a 6 mL  min^−1^ flow rate and a linear gradient over 30 min of 15−60% of solvent B/A for PHP* and 5−60% B/A for PHP (A: 0.1% TFA in H_2_O, B: 0.1% TFA in CH_3_CN). Peptides were monitored at 220 and 330 nm (the latter for TNP moiety detection). Peptide purity was assessed with analytical reverse‐phased HPLC (RP‐C18 column, 5 µm, 100 × 4.6 mm, Higgins Analytical) with a 1 mL min^−1^ flow rate and a linear gradient over 30 min of 15−60% of solvent B in A for PHP* and 5−60% B in A for PHP (Figure S2, Supporting Information). The peptide were characterized by matrix‐assisted laser desorption‐ionization time‐of‐flight (MALDI‐TOF) mass spectrometry (Applied Biosystems 4700 or AB Sciex Voyager DE‐PRO spectrometer) in positive‐ion mode, linear, with CHCA (α‐cyano‐4‐hydroxycinnamic acid) at 10 mg mL^−1^ in H_2_O/CH_3_CN/TFA (1: 1: 0.01) as matrix (Table S2; Figure S2, Supporting Information).

### Antibodies Reduction

Partial reduction of anti‐HSP27 fluorescent antibody was conducted by mixing Ab stock solution [2 mg mL^−1^ in dPBS] (20 µL, 1 mg mL^−1^) with EDTA [stock solution: 50 mm in H_2_Od (distilled water), pH 8] (8 µL, 10 mm), sodium phosphate [stock solution: 0.5 m in H_2_Od, pH 8] (8 µL, 100 mm) and sodium chloride [stock solution: 1.5 m in H_2_Od, pH 8] (4 µL, 150 mm). TCEP [5 µM in H_2_Od] was added (266.8 µL, 10 eq). The mixture was stirred 90 min at 37 °C, 480 rpm, and washed in nanosep columns 10 kDa (Pall Corporation, France) with dPBS (3 × 5 min, 9000 x g) and concentrated to obtain a final volume of 80 µL (0.25 mg mL^−1^).

For SDS‐Page analysis, polyacrylamide resolving gels of 7% in acrylamide were prepared by mixing 2.5 mL of tris(hydroxymethyl)aminoethane [1.5 m, pH 8.8], with 4.99 mL of milliQ water, 2.33 mL of acrylamide/bisacrylamide 30%, 0.1 mL of SDS 10%, 70 µL of ammonium persulfate 10% (in weight) and 10 µL of tetramethylethylenediamine. This solution was hand mixed and poured between two glasses plate with spacers of 1 mm. Once polymerized, a stacking gel with 4.5% in acrylamide was prepared by mixing 1.25 mL of tris(hydroxymethyl)aminoethane [0.5 m, pH 6.8], 2.9 mL of milliQ water, 0.75 mL of acrylamide/bisacrylamide 30%, 50 µL of SDS 10%, 35 µL of ammonium persulfate 10% (in weight) and 5 µL of tetramethylethylenediamine. A 10 wells comb was inserted before polymerization. One volume of sample (5 µg of Ab) was mixed with a quarter of this volume of 5X Laemmli buffer (2.5 mL of tris(hydroxymethyl)aminoethane [0.5 m, pH 6.8], 40% (v/v) of glycerol, 6.25% (w/v) of SDS and 0.1% (w/v) of bromophenol blue. These solutions were heated 10 min at 95 °C and loaded onto the wells. 3 µL of PageRuler Plus prestained protein ladder were also loaded into the first well. For migration, a constant current of 25 mA was applied, and switched to 35 mA once the migration front entered the resolving gel. The final gel was silver stained using the protocol developed by Blum *et al.* ^[^ ^53^ ^]^ Images were taken with the Chemidoc Imaging System and analyzed with Image Lab software.

### Nanoparticles Synthesis

Maghemite nanoparticles γ‐Fe_2_O_3_ with an average physical diameter d = 11.3 nm (σ = 0.3) were obtained by alkaline co‐precipitation of iron salts. Iron(II) chloride tetrahydrate (FeCl_2_.4H_2_O) (180 g, 0.905 mol) was dissolved in HCl 37% (100 mL) and H_2_O (500 mL). The mixture was poured in iron(III) chloride hexahydrate (FeCl_3_.6H_2_O) (715 mL, 1.59 mol) and agitated before adding ammonia 22% (1 L). The mixture was stirred for 30 min at RT. NPs were magnetically separated from the solution and washed with deionized H_2_O. NPs were acidified with nitric acid 52.5% (360 mL) in H_2_O (1.64 L) and agitated at RT for 30 min before being magnetically separated from the solution. A solution of iron(III) nitrate nonahydrate (Fe(NO_3_)_3_.9H_2_O) (323 g, 0.80 mol) previously dissolved in H_2_O (800 mL) and heated at 180 °C was added to the pellet to oxidize magnetite particles. The mixture was stirred and heated at 30 °C for 30 min. After phase separation on a magnet, maghemite particles were acidified with nitric acid 52.5% (360 mL) in H_2_O (2 L). The mixture was agitated 10 min at RT, the particles were magnetically separated from the solution and dispersed in H_2_O (500 mL). Obtained particles were size sorted by phase separation on a magnet after addition of nitric acid (2 × 20 mL). Precipitated particles were washed with acetone (3 × 600 mL) and diethyl ether (2 × 400 mL) and dispersed in 200 mL H_2_O. Sodium citrate dihydrate (Na_3_C_6_H_5_O_7_.2H_2_O) (7.82 g, 26.60 mmol) was added to 150 mL of the fraction. The mixture was stirred and heated at 100 °C for 30 min, then washed with acetone (3 × 600 mL) and diethyl ether (2 × 400 mL). NPs were finally dispersed in H_2_O (150 mL). This suspension exhibited a final iron concentration of 1.3 mol L^−1^.

Thiol‐coated core‐shell (CS‐SH) synthesis was achieved from maghemite nanoparticles solution (50 µL, 65 µmol) in H_2_O/EtOH 1: 2 (15 mL). The silica shell was obtained by condensation of silylated precursors in alkaline medium by adding TEOS (56 µL, 0.25 mmol) with ammonia 30% (250 µL, 3.90 mmol) to the reaction mixture. For fluorescent CS‐SH (CS*‐SH), rhodamine‐B isothiocyanate previously functionalized with APTS (Rhod‐APTS) was added in this first step (18.6 µL, 0.076 µmol). The mixture was agitated for 2 h at RT and degassed for 15 min. Silica shell was then functionalized by adding TEOS (19.55 µL, 90 µmol), ZSSi [100 mg mL^−1^ in H_2_O] (123.75 µL, 37.6 µmol), and MPTS [41.4 mM in EtOH] (34 µL, 1.41 µmol) along with TCEP 0.5 M (28.59 µL, 14.1 µmol). The mixture was stirred 15 h at RT. CS were washed with diethyl ether: EtOH (15: 1, 3 × 30 mL) and dispersed in 5 mL of MOPS buffer (0.1 m, pH 7.5). Final suspension exhibited an iron concentration of 16,2 mm (Table S1, Supporting Information). For further surface functionalization of these CS‐SH particles, thiol functions introduced with MPTS were used as the 1 eq. Reference.

### CS‐SH Surface Functionalization

PHP* immobilization on CS*‐SH surface: PHP* immobilization at the surface of NPs was conducted by mixing CS*‐SH solution (100 µL, 28.6 nmol of thiols introduced with MPTS) with PHP* [1 mM in H_2_O] (85.68 µL, 3 eq.) o/n at RT. CS*‐PHP* nanoparticles were washed using ultrafiltration columns Amicon Ultra, 100 kDa with HEPES (200 mm, pH 7.5)/EDTA (2 mM) (3×5 min, 4000 x g) and HEPES (200 mM, pH 7.5) (2×5 min, 4000 x g) to eliminate the remaining EDTA. Nanoparticles were dispersed in HEPES (200 mm, pH 7.5) to obtain a total volume of 100 µL.

Fluorescein‐maleimide (FAM) functionalized CS*‐SH (CS*‐FAM): Thiol‐coated CS* solution (100 µL, 28.6 nmol) was mixed with fluorescein‐maleimide (FAM) solution [1 mm in DMSO] (14.3 µL, 0.5 eq.) for 2 h at RT. CS*‐FAM nanoparticles were washed using ultrafiltration columns Amicon Ultra, 100 kDa with HEPES (200 mm, pH 7.5)/EDTA (2 mM) (3 × 5 min, 4000 x g) and HEPES (200 mm, pH 7.5) (2 × 5 min, 4000 x g) to eliminate the remaining EDTA. Nanoparticles were redispersed in HEPES (200 mm, pH 7.5) to obtain a total volume of 100 µL.

Functionalization of CS with fluorescent probes and PHP (CS*‐FAM‐PHP and CS‐ROX‐PHP*): PHP immobilization onto the surface of fluorescent CS was conducted by mixing CS*‐FAM or CS‐ROX solution (100 µL) with PHP or PHP* [1 mM in H_2_O] (14.3 µL, 0.5 eq.) for 24 h at RT. CS*‐FAM‐PHP or CS‐ROX‐PHP* nanoparticles were washed using ultrafiltration columns Amicon Ultra, 100 kDa with HEPES (200 mm, pH 7.5)/EDTA (2 mM) (3 × 5 min, 4000 x g) and HEPES (200 mm, pH 7.5) (2 × 5 min, 4000 x g) to eliminate the remaining EDTA. Nanoparticles were redispersed in HEPES (200 mm, pH 7.5) to obtain a total volume of 100 µL.

Thiol‐coated CS activation with BMB (CS*‐BMB): Thiol‐coated CS (CS*‐SH) solution (200 µL, 57.2 nmol of SH) was mixed with BMB [2 mM in DMSO] (57.2 µL, 2 eq) at 480 rpm, RT, for 2 h. CS*‐BMB nanoparticles were washed using ultrafiltration columns Amicon Ultra, 100 kDa with HEPES (200 mm, pH 7.5)/EDTA (2 mM) (3 × 5 min, 4000 x g) and HEPES (200 mm, pH 7.5) (2 × 5 min, 4000 x g) to eliminate the remaining EDTA.

Antibodies grafting onto CS surface (CS*‐Ab*): 100 µL of CS*‐BMB (28.6 nmol of BMB) were mixed with the previously reduced antibody (1, 2 or 3 Ab*/CS*, 5.7 µL, 11.4 µL or 17.1 µL respectively) and agitated at 480 rpm, at 37 °C for 1 h. CS*‐Ab* particles were washed using ultrafiltration columns Vivaspin, 300 kDa (Sartorius, Germany) with HEPES (200 mm, pH 7.5)/EDTA (2 mm) (3 × 5 min, 2200 x g) and HEPES (200 mm, pH 7.5) (2 × 5 min, 2200 x g) to eliminate the remaining EDTA, and resuspended in HEPES to obtain a final volume of 100 µL.

Bi‐functionalized CS synthesis (CS*‐PHP‐Ab*): Thiol‐coated CS* (50 µL, 14.3 nmol of SH) were mixed with polyhistidine peptides [1 mM in H_2_O] (fluorescent or not) (7.15 µL, 0.5 eq) and agitated at 480 rpm overnight. Then, particles were washed using ultrafiltration columns Amicon Ultra, 100 kDa with HEPES (200 mm, pH 7.5)/EDTA (2 mm) (3 × 5 min, 4000 xg) and HEPES (200 mm, pH 7.5) (2 × 5 min, 4000 xg), and dispersed on HEPES to obtain a final volume of 50 µL. This solution was mixed with BMB [2 mM in DMSO] (7.15 µL, 2 eq) for 2 h at RT at 480 rpm and washed following the same protocol. Eventually, this solution was mixed with the previously reduced Ab* to graft 1 or 3 Ab*/CS*. CS*‐PHP‐Ab* particles were washed using ultrafiltration columns Vivaspin 300 kDa (Sartorius, Germany) with HEPES (200 mm, pH 7.5)/EDTA (2 mm) (3 × 5 min, 2200 x g) and HEPES (200 mm, pH 7.5) (2 × 5 min, 2200 x g), and resuspended in HEPES to obtain a final volume of 50 µL.

### Nanoparticle Characterization

Iron concentration: Iron concentration of resulting nanoparticles was estimated by atomic absorption spectroscopy at 248 nm (PerkinElmer PinAAcle 500). MNPs were dissolved in HCl 37% and diluted in HNO_3_ 2%.

### Hydrodynamic Diameter and Zeta Potential

MNPs stability and hydrodynamic diameter were monitored by dynamic light scattering (ZetaSizer Ultra, Malvern, United Kingdom, detection angle: 173°, laser wavelength: 656 nm) in H_2_O at 25 °C (viscosity: 0.887 cP; refractive index: 3.13; absorbance: 0.071) or in cell medium DMEM/F12 at 37 °C (viscosity: 0.686 cP; refractive index: 1.33; absorbance: 0.071). The zeta potential ζ of the particles at pH 6 and at 25 °C was obtained by Zetametry.

### Transmission Electron Microscopy

Nanoparticles size and shape were evaluated by transmission electron microscopy (TEM). A droplet of diluted NPs in H_2_O was placed on a carbon‐coated copper grill and let to dry. NPs were then characterized using a JEOL‐1011 transmission electron microscope. The average physical size was calculated with a lognormal fit on the size distribution. Images were analyzed with ImageJ by contouring each particle. The software then gives an area that is used to calculate the diameter by assuming that the particle is spherical.

### Fluorescence Spectroscopy

Fluorescence profiles were recorded by placing samples on black 96‐well plates (FAM: λ_exc_ = 494 nm, λ_em_ = 520 nm, ROX: λ_exc_ = 570 nm, λ_em_ = 591 nm, rhodamine‐B in silica shell: λ_exc_ = 540 nm, λ_em_ = 585 nm, CF: λ_exc_ = 493 nm, λ_em_ = 517 nm) (SpectraMax i3x, Molecular Devices, US).

### Cleavage Assay with Glutathione

CS*‐PHP* nanoparticles (45 µL) were mixed with a glutathione solution (5 µL, 100 mm) for 0, 1, 3, 5, and 24 h at RT. Samples were washed using ultrafiltration columns Amicon Ultra, 100 kDa with HEPES (200 mm, pH 7.5)/EDTA (2 mm) (3 × 5 min, 4000 x g) and HEPES (200 mm, pH 7.5) (2 × 5 min, 4000 x g). Nanoparticles were redispersed in HEPES (200 mm, pH 7.5) to obtain a total volume of 50 µL. Solutions were placed on a black 96‐well plate to record rhodamine fluorescence (λ_exc_ = 540 nm, λ_em_ = 585 nm) and CF fluorescence (λ_exc_ = 493 nm, λ_em_ = 517 nm) (SpectraMax i3x, Molecular Devices, US).

### Cells Studies

Cell culture: SH‐SY5Y cells were cultured in Dulbecco's modified eagle medium/nutrient mixture F‐12 50: 50 (DMEM/F12) supplemented with 10% of heat inactivated fetal bovine serum (HI‐FBS) and 1% of a penicillin (100 000 IU L^−1^) and streptomycin (100 000 IU L^−1^) mixture. Cells were incubated in humidified atmosphere with 5% CO_2_ at 37 °C.

Cells transfection with pEGFP‐HSP27: Plasmid DNA was purified from stab culture of bacteria. A single bacterial colony was isolated from a streaked Luria broth (LB) agar plate, containing Kanamycin antibiotic (50 µg mL^−1^) and cultured overnight in liquid LB. Plasmid DNA was recovered from this bacterial culture with the GeneJET Plasmid Miniprep Kit and the obtained plasmid DNA was stored at ‐20 °C. SH‐SY5Y cell line was transfected with the plasmid DNA following the Lipofectamine 3000 protocol.

Cytotoxicity measurement with LDH assay: On a 96‐well plate, 10 000 cells were seeded per well and let grow for 48 h at 37 °C in humidified atmosphere with 5% CO_2_. CS samples were then incubated at different concentrations in triplicate wells (at iron concentrations 0.125, 0.25, 0.5, and 1 mm) in DMEM/F12, 50 µL, for 6 h at 37 °C in humidified atmosphere with 5% CO_2_. 50 µL of supplemented cell culture medium were added. After 24 h of incubation, 10 µL of lysis buffer were added in control wells and incubated for 30 min. 50 µL of each well were transferred into a new 96‐well plate and 50 µL of reaction mixture were added in each well and incubated for 30 min at RT protected from light. 50 µL of Stop solution were added to each sample well and bubbles were removed with a syringe needle. Absorbance was recorded at 490 and 680 nm. To determine LDH activity, the 680 nm absorbance values were subtracted from the 490 nm absorbance values. The percentage of cytotoxicity was calculated using the following formula:% Cytotoxicity = [(Compound treated LDH activity‐Spontaneous LDH activity)/(Maximum LDH activity‐Spontaneous LDH activity)]×100

### Internalized Peptides Quantification

PHP* fluorescence standard curve was generated by preparing a range of solutions exhibiting different PHP* amounts from 0 pmoles to 0.12 pmoles in lysis buffer (Tris 50 mm (pH 7.4), NaCl 1 m, NP40 1%, TCEP 0.1 m, V_tot_ = 300 µL), in the presence of 1 million cells. After 15 min incubation, samples were centrifuged for 5 min (900 x g). Supernatants were collected and PHP* fluorescence was recorded in black 96‐well plate (SpectraMax i3x, Molecular Devices, US; λ_exc_ = 493 nm, λ_em_ = 517 nm). Samples were diluted to avoid quenching of fluorescence.

To assess CS‐PHP* internalization referring to this standard curve, cells were seeded on a 12‐well plate and let grow for 48 h to reach 1 million cells and incubated with CS‐PHP* at [Fe] = 1 mm for 4 h and washed. 20 h later, cells were then treated with TCEP in HBSS (5 mM) for 15 min and washed with HBSS (1 × 300 µL). Cells were treated with trypsin/EDTA (0.25%/0.02%) for 3 min (500 µL). Trypsin activity was blocked adding soybean inhibitor (5 mg mL^−1^ in PBS, 100 µL) and BSA (1 mg mL^−1^, 100 µL). Cells were transferred in microtubes and wells were rinsed with 300 µL of Tris buffer (50 mm, pH 7.4). Cells were counted 3 times. Cells were centrifuged for 5 min (900 x g) and the supernatant was eliminated. Cells were washed with 1 mL of Tris buffer 50 mM, pH 7.4, 0.1% BSA. Lysis buffer Tris 50 mm (pH 7.4), NaCl 1 m, NP40 1%, TCEP 0.1 m was added to cells (300 µL, 15 min) and they were centrifuged for 5 min (900 x g). For quantification of the peptide associated to cells (internalized and membrane‐bound peptides), treatment with TCEP/trypsin/EDTA was omitted and cells were directly lysed. Supernatant was collected and its fluorescence emission was measured. As for the standard solutions, samples were diluted to avoid quenching of fluorescence and to have values included in the standard curve.

### Flow Cytometry

Cells were seeded on a 12‐well plate and let grow for 48 h to reach 1 million cells and incubated with CS‐PHP* at [Fe] = 1 mm for 4 h and washed. 20 h later, cells were treated with TCEP in HBSS (5 mm) for 15 min and washed with HBSS (1 × 300 µL). Cells were treated with 500 µL of either trypsin/EDTA (0.25%/0.02%, 37 °C, 5 min), or pronase (0.05% in Tris buffer 100 mm, pH 7.4, 4 °C, 10 min). Trypsin activity was blocked adding soybean inhibitor (5 mg mL^−1^ in PBS, 100 µL) and BSA (1 mg mL^−1^, 100 µL), and pronase activity was blocked at 4 °C with 100 µL of a pronase inhibitor (one Complete‐Mini tablet, Roche in 2.5 mL H_2_Od) and BSA (1 mg mL^−1^, 100 µL). Cells were transferred in microtubes and wells were rinsed with 300 µL of Tris buffer (50 mm, pH 7.4). Cells were centrifuged for 5 min (900 x g) and supernatant was eliminated. Cells were washed with 1 mL of Tris buffer 50 mm, pH 7.4, 0.1% BSA and they were centrifuged for 5 min (900 x g). 400 µL of PBS was added to cells. For half of the cell sample, fluorescence was measured in PBS and the other half of sample was treated with blue trypan (0.2% final concentration) to verify that all the membrane bound was digested par trypsin or pronase. Fluorescence emission was measured by flow cytometry using a FACSCalibur (BD Biosciences) and analyzed by the BD CellQuestTM software. Wavelengths were 488 nm for excitation and 525 nm for detection, and fluorescence was measured one more time. For each condition, 20 000 events were measured for each condition.

### Internalized Nanoparticles Imaging on Cells

Cells were seeded onto Ibidi µ‐Slide 8 Well (10 000 cells/well) with supplemented DMEM/F12 (total volume = 150 µL) and let grow for 48 h at 37 °C in humidified atmosphere with 5% CO_2_. Cells were then washed with 150 µL DMEM/F12 and samples were incubated at [Fe] = 1 mm for 4 h with DMEM/F12 before being washed twice using supplemented DMEM/F12 and samples were observed after 6 h, 24 h, or 48 h. Just before observation, cells were washed twice with DMEM/12 and three times with HBSS. Cell nuclei were dyed with Hœchst 33342 dye for 15 min. Cells were washed one last time with HBSS and 200 µL of HBSS were finally added. When needed, trypan blue 0.4% were added in each well just before observation (75 µL, 0.1%). Live cells were imaged on a Leica SP5 confocal microscope using a 63X oil immersion objective lens, with pinhole aperture set to 1 Airy unit and a z‐step of 130 nm. Final images were generated as an average intensity projection of ten z‐stacked images (final image z = 1.3 µm) using FIJI ImageJ.

### Colocalization Analysis

For Manders’ coefficients calculation, the JACoP plugin was used on FIJI ImageJ, “image A” was selected to be the one with red fluorescence and “image B” the one with green fluorescence. Statistical significance was determined using the DiAna plugin on FIJI ImageJ and a spot segmentation was applied.

## Conflict of Interest

The authors declare no conflict of interest.

## Supporting information



Supporting Information

## Data Availability

The data that support the findings of this study are available from the corresponding author upon reasonable request.

## References

[1] N. V. Long , Y. Yang , T. Teranishi , C. M. Thi , Y. Cao , M. Nogami , J. Nanosci. Nanotechnol. 2015, 15, 10091. 26682455 10.1166/jnn.2015.11691

[2] V. F. Cardoso , A. Francesko , C. Ribeiro , M. Bañobre‐López , P. Martins , S. Lanceros‐Mendez , Adv. Healthcare Mater. 2018, 7, 1700845. 10.1002/adhm.201700845 29280314

[3] U. Jeong , X. Teng , Y. Wang , H. Yang , Y. Xia , Adv. Mater. 2007, 19, 33.

[4] N. A. Frey , S. Peng , K. Cheng , S. Sun , Chem. Soc. Rev. 2009, 38, 2532. 19690734 10.1039/b815548h PMC2740941

[5] C. Monzel , C. Vicario , J. Piehler , M. Coppey , M. Dahan , Chem. Sci. 2017, 8, 7330. 29163884 10.1039/c7sc01462g PMC5672790

[6] S. Del Sol‐Fernández , P. Martínez‐Vicente , P. Gomollón‐Zueco , C. Castro‐Hinojosa , L. Gutiérrez , R. M. Fratila , M. Moros , Nanoscale 2022, 14, 2091. 35103278 10.1039/d1nr06303k PMC8830762

[7] F. Raudzus , H. Schöneborn , S. Neumann , E. Secret , A. Michel , J. Fresnais , O. Brylski , C. Ménager , J.‐M. Siaugue , R. Heumann , Sci. Rep. 2020, 10, 22452. 33384447 10.1038/s41598-020-80253-w PMC7775457

[8] H. Schöneborn , F. Raudzus , E. Secret , N. Otten , A. Michel , J. Fresnais , C. Ménager , J.‐M. Siaugue , H. Zaehres , I. D. Dietzel , R. Heumann , J. Funct. Biomater. 2019, 10, 32. 31315182 10.3390/jfb10030032 PMC6787644

[9] V. I. P. Keizer , S. Grosse‐Holz , M. Woringer , L. Zambon , K. Aizel , M. Bongaerts , F. Delille , L. Kolar‐Znika , V. F. Scolari , S. Hoffmann , E. J. Banigan , L. A. Mirny , M. Dahan , D. Fachinetti , A. Coulon , Science 2022, 377, 489. 35901134 10.1126/science.abi9810

[10] S. D. Conner , S. L. Schmid , Nature 2003, 422, 37. 12621426 10.1038/nature01451

[11] R. Di Corato , A. Espinosa , L. Lartigue , M. Tharaud , S. Chat , T. Pellegrino , C. Ménager , F. Gazeau , C. Wilhelm , Biomaterials 2014, 35, 6400. 24816363 10.1016/j.biomaterials.2014.04.036

[12] F. Mazuel , A. Espinosa , N. Luciani , M. Reffay , R. Le Borgne , L. Motte , K. Desboeufs , A. Michel , T. Pellegrino , Y. Lalatonne , C. Wilhelm , ACS Nano 2016, 10, 7627. 27419260 10.1021/acsnano.6b02876

[13] A. P. Sangnier , A. B. Van de Walle , A. Curcio , R. L. Borgne , L. Motte , Y. Lalatonne , C. Wilhelm , Nanoscale 2019, 11, 16488. 31453605 10.1039/c9nr05624f

[14] T. Bus , A. Traeger , U. S. Schubert , J. Mater. Chem. B 2018, 6, 6904. 32254575 10.1039/c8tb00967h

[15] E. C. Freeman , L. M. Weiland , W. S. Meng , J. Biomater. Sci., Polym. Ed. 2013, 24, 398. 23565683 10.1080/09205063.2012.690282 PMC3623018

[16] L. M. P. Vermeulen , S. C. De Smedt , K. Remaut , K. Braeckmans , Eur. J. Pharm. Biopharm. 2018, 129, 184. 29859281 10.1016/j.ejpb.2018.05.034

[17] M. Omura , K. Morimoto , Y. Araki , H. Hirose , Y. Kawaguchi , Y. Kitayama , Y. Goto , A. Harada , I. Fujii , T. Takatani‐Nakase , S. Futaki , I. Nakase , ACS Appl. Mater. Interfaces 2023, 15, 47855. 37792057 10.1021/acsami.3c01650 PMC10592309

[18] Q. Chen , Q. Qian , H. Xu , H. Zhou , L. Chen , N. Shao , K. Zhang , T. Chen , H. Tian , Z. Zhang , M. Jones , K. Y. H. Kwan , M. Sewell , S. Shen , X. Wang , M. A. Khan , P. Makvandi , S. Jin , Y. Zhou , A. Wu , ACS Nano 2024, 18, 8885. 38465890 10.1021/acsnano.3c12163

[19] T. Kim , H. S. Han , K. Yang , Y. M. Kim , K. Nam , K. H. Park , S. Y. Choi , H. W. Park , K. Y. Choi , Y. H. Roh , ACS Nano 2024, 18, 7972. 38445578 10.1021/acsnano.3c10732

[20] S. Narum , B. Deal , H. Ogasawara , J. N. Mancuso , J. Zhang , K. Salaita , ACS Nano 2024, 18, 6186. 38346399 10.1021/acsnano.3c09027 PMC10906071

[21] R. Ray , S. Ghosh , A. Maity , N. R. Jana , ACS Appl. Mater. Interfaces 2024, 16, 5451. 38265005 10.1021/acsami.3c14472

[22] F. Etoc , E. Balloul , C. Vicario , D. Normanno , D. Liße , A. Sittner , J. Piehler , M. Dahan , M. Coppey , Nat. Mater. 2018, 17, 740. 29967464 10.1038/s41563-018-0120-7

[23] Z. Wang , J. Zhang , Y. Wang , J. Zhou , X. Jiao , M. Han , X. Zhang , H. Hu , R. Su , Y. Zhang , W. Qi , ACS Nano 2024, 18, 10324. 38547369 10.1021/acsnano.4c02400

[24] L. Yu , Y. Xu , M. Al‐Amin , S. Jiang , M. Sample , A. Prasad , N. Stephanopoulos , P. Šulc , H. Yan , J. Am. Chem. Soc. 2023, 145, 27336. 38055928 10.1021/jacs.3c07491 PMC10789493

[25] H. Y. Zhao , Y. Q. Chen , X. Y. Luo , M. J. Cai , J. Y. Li , X. Y. Lin , H. Zhang , H. M. Ding , G. L. Jiang , Y. Hu , ACS Nano 2024, 18, 2162. 38198577 10.1021/acsnano.3c09452

[26] M. Le Jeune , E. Secret , M. Trichet , A. Michel , D. Ravault , F. Illien , J.‐M. Siaugue , S. Sagan , F. Burlina , C. Ménager , ACS Appl. Mater. Interfaces 2022, 14, 15021. 35319860 10.1021/acsami.2c01346

[27] K. L. Williams , M. Rahimtula , K. M. Mearow , BMC Neurosci. 2005, 6, 24. 15819993 10.1186/1471-2202-6-24 PMC1087488

[28] D. A. Bechtold , I. R. Brown , Neurochem. Res. 2003, 8, 1163. 10.1023/a:1024268126310 12834255

[29] M. P. Monopoli , C. Aberg , A. Salvati , K. A. Dawson , Nat. Nanotechnol. 2012, 7, 779. 23212421 10.1038/nnano.2012.207

[30] F. Etoc , C. Vicario , D. Lisse , J.‐M. Siaugue , J. Piehler , M. Coppey , M. Dahan , Nano Lett. 2015, 15, 3487. 25895433 10.1021/acs.nanolett.5b00851

[31] H. J. Forman , H. Zhang , A. Rinna , Mol. Aspects Med. 2009, 30, 1. 18796312 10.1016/j.mam.2008.08.006 PMC2696075

[32] H. Li , J. Han , G. Liang , ACS Appl. Nano Mater. 2020, 3, 1489.

[33] M. Debayle , E. Balloul , F. Dembele , X. Xu , M. Hanafi , F. Ribot , C. Monzel , M. Coppey , A. Fragola , M. Dahan , T. Pons , N. Lequeux , Biomaterials 2019, 219, 119357. 31351245 10.1016/j.biomaterials.2019.119357

[34] S. El Mousli , Y. Dorant , E. Bertuit , E. Secret , J.‐M. Siaugue , J. Magn. Magn. Mater. 2024, 589, 171571.

[35] G. T. Kozma , T. Shimizu , T. Ishida , J. Szebeni , Adv. Drug Delivery Rev. 2020, 154, 163. 10.1016/j.addr.2020.07.024 32745496

[36] Z. Amoozgar , Y. Yeo , W. Interdiscip. Rev. Nanomed. Nanobiotechnol. 2012, 4, 219. 10.1002/wnan.1157 PMC3288878 22231928

[37] R. Massart , E. Dubois , V. Cabuil , E. Hasmonay , J. Magn. Magn. Mater. 1995, 149, 1.

[38] A. Burns , P. Sengupta , T. Zedayko , B. Baird , U. Wiesner , Small 2006, 2, 723. 17193111 10.1002/smll.200600017

[39] E. M. M. Manders , J. Stap , G. J. Brakenhoff , R. V. Driel , J. A. Aten , J. Cell Sci. 1992, 103, 857. 1478975 10.1242/jcs.103.3.857

[40] S. Bolte , F. P. Cordelières , J. Microsc. 2006, 224, 213. 17210054 10.1111/j.1365-2818.2006.01706.x

[41] J.‐F. Gilles , M. Dos Santos , T. Boudier , S. Bolte , N. Heck , Methods 2017, 115, 55. 27890650 10.1016/j.ymeth.2016.11.016

[42] S. Saidjalolov , F. Coelho , V. Mercier , D. Moreau , S. Matile , ACS Cent. Sci. 2024, 10, 1033. 38799667 10.1021/acscentsci.3c01601 PMC11117725

[43] J.‐M. Swiecicki , F. Thiebaut , M. Di Pisa , S. Gourdin‐Bertin , J. Tailhades , C. Mansuy , F. Burlina , S. Chwetzoff , G. Trugnan , G. Chassaing , S. Lavielle , Sci. Rep. 2016, 6, 20237. 26839211 10.1038/srep20237 PMC4738315

[44] F. Illien , N. Rodriguez , M. Amoura , A. Joliot , M. Pallerla , S. Cribier , F. Burlina , S. Sagan , Sci. Rep. 2016, 6, 36938. 27841303 10.1038/srep36938 PMC5107916

[45] S. Aubry , F. Burlina , E. Dupont , D. Delaroche , A. Joliot , S. Lavielle , G. Chassaing , S. Sagan , FASEB J. Off. Publ. Fed. Am. Soc. Exp. Biol. 2009, 23, 2956. 10.1096/fj.08-127563 19403512

[46] M. M. C. Sun , K. S. Beam , C. G. Cerveny , K. J. Hamblett , R. S. Blackmore , M. Y. Torgov , F. G. M. Handley , P. D. Senter , S. C. Alley , Bioconjug. Chem. 2005, 16, 1282. 16173809 10.1021/bc050201y PMC2539111

[47] S. Yoshitake , Y. Yamada , E. Ishikawa , R. Masseyeff , Eur. J. Biochem. 1979, 101, 395. 574817 10.1111/j.1432-1033.1979.tb19731.x

[48] S. Jeong , J. Y. Park , M. G. Cha , H. Chang , Y. Kim , H.‐M. Kim , B.‐H. Jun , D. S. Lee , Y.‐S. Lee , J. M. Jeong , Y.‐S. Lee , D. H. Jeong , Nanoscale 2017, 9, 2548. 28150822 10.1039/c6nr04683e

[49] R. Guo , H. Peng , Y. Tian , S. Shen , W. Yang , Small 2016, 12, 4541. 27390093 10.1002/smll.201601094

[50] Y. Zhang , Y. Shen , X. Teng , M. Yan , H. Bi , P. C. Morais , ACS Appl. Mater. Interfaces 2015, 7, 10201. 25942702 10.1021/acsami.5b00405

[51] J. Choi , J. Shin , J. Lee , M. Cha , Chem. Commun. 2012, 48, 7474. 10.1039/c2cc33659f 22728544

[52] O. H. Voss , S. Batra , S. J. Kolattukudy , M. E. Gonzalez‐Mejia , J. B. Smith , A. I. Doseff , J. Biol. Chem. 2007, 282, 25088. 17597071 10.1074/jbc.M701740200

[53] H. Blum , H. Beier , H. J. Gross , Electrophoresis 1987, 8, 93.

