# Supplementary material for: Intracellular Proteins Targeting with Bi‐Functionalized Magnetic Nanoparticles Following their Endosomal Escape
Source: Small. 2025 Feb 19;21(13):2410454. doi: 10.1002/smll.202410454 (PMC11962688; doi:10.1002/smll.202410454)
Supplement: Supplementary file 1 — Supporting Information [file SMLL-21-2410454-s001.pdf]

NANO · MICRO
small

Supporting Information

for *Small*, DOI 10.1002/smll.202410454

Intracellular Proteins Targeting with Bi-Functionalized Magnetic Nanoparticles Following their Endosomal Escape

*Mélody Perret, Estelle Pineda, Mathilde Le Jeune, Tieu Ngoc Nguyen, Aude Michel, Françoise Illien, Jean-Michel Siaugue, Christine Ménager, Fabienne Burlina and Emilie Secret**

Supporting Information

Intracellular proteins targeting with bi-functionalized magnetic nanoparticles following their endosomal escape

*Mélody Perret, Estelle Pineda, Mathilde Le Jeune, Tieu Ngoc Nguyen, Aude Michel, Françoise Illien, Jean-Michel Siaugue, Christine Ménager, Fabienne Burlina, Emilie Secret**

- **Thiol-coated CS synthesis**

Table S1. MNP iron concentrations determined by atomic absorption spectroscopy (AAS), hydrodynamic diameter (averaged in number), $Z_{average}$ and zeta potential.

Sample	Iron concentration (mM) [a]	Hydrodynamic diameter in number (nm) [b]	$Z_{average}$ (nm)	PDI	Zeta Potential (mV)[b]
CS*-SH	16.2 ± 0.1	39.6	98.6	0.3	- 47.5
CS*-FAM	11.4 ± 0.3	38.6	89.2	0.2	- 43.9
CS*-FAM-PHP	9.5 ± 0.1	31.6	84.7	0.4	- 43.8
CS*-PHP*	13.1 ± 0.1	31.3	85.5	0.4	- 41.8

[a] Mean : (n=3)
[b] Zeta potential and hydrodynamic size distributions are shown in Fig. S1 and Fig. S3.

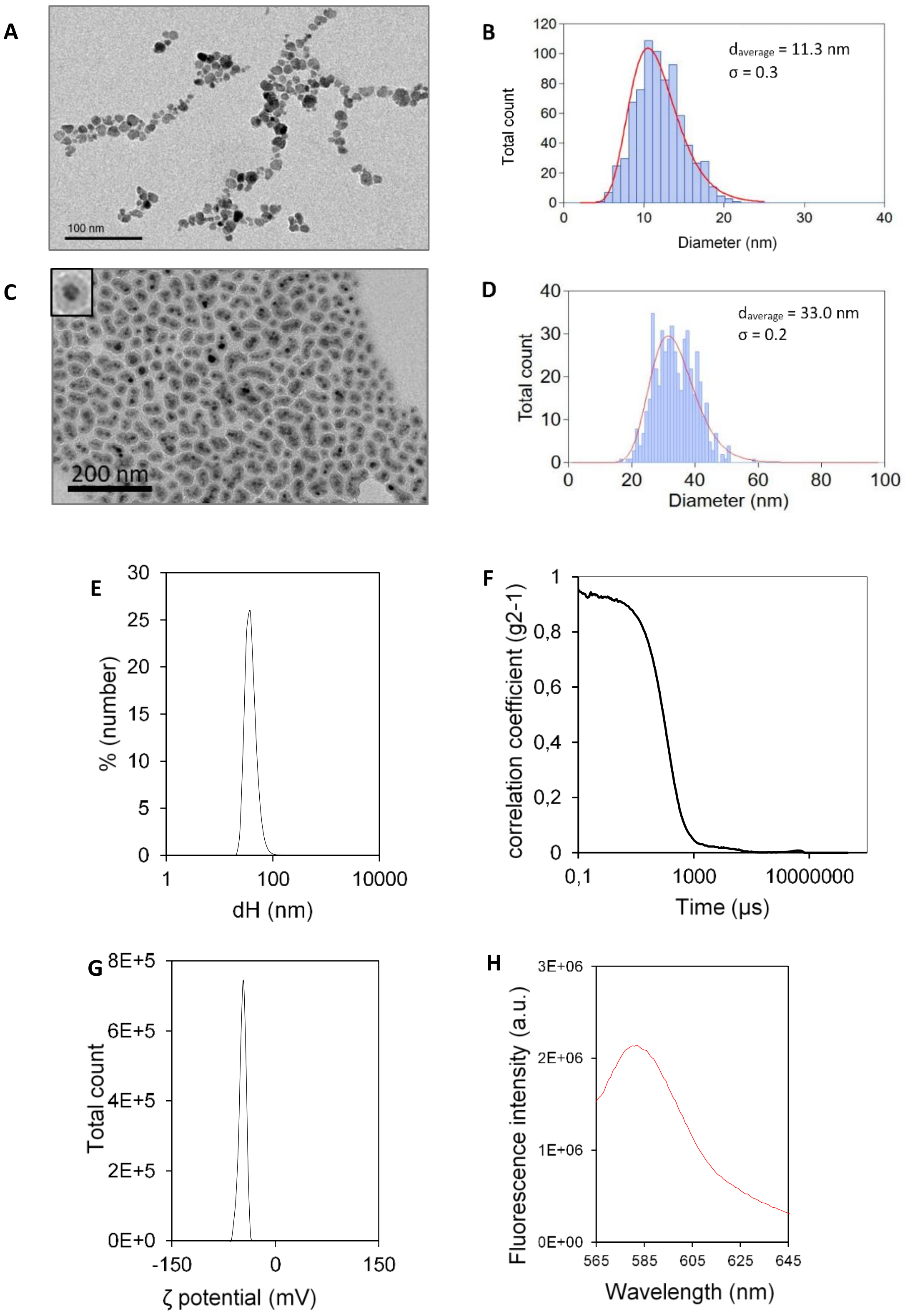

Figure S1. (A-D) Characterization of MNPs with transmission electron microscopy (TEM) : (A) TEM image of iron oxide nanoparticles obtained by alkaline co-precipitation of iron salts and (B) corresponding size distribution (measured on 300 particles). (C) TEM image of CS*-

SH and (D) corresponding size distribution (measured on 500 particles). (E-H) Characterization of CS*-SH nanoparticles: (E) Hydrodynamic diameter determined with DLS, (F) DLS correlation function, (G) ζ-potential determined with zetametry and (G) Rhodamine fluorescence spectrum of CS*-SH (λ_{exc} = 540 nm, λ_{em} = 585 nm).

- **<u>Peptide-functionalized CS synthesis</u>**

Polyhistidine peptides characterization:

Table S2. Corresponding m/z values from figure S6 for fluorescent and non-fluorescent peptides analyzed with MALDI-TOF mass spectroscopy.

Fluorescent peptide (PHP*)			Non fluorescent peptide (PHP)		
**Expected m/z**	**Observed m/z**	**Corresponding ion**	**Expected m/z**	**Observed m/z**	**Corresponding ion**
1326.48	1326.47	[M^+ w/o TNP]	855.20	855.90	[MH^+ w/o TNP]
1349.47	1349.67	[MNa^+ w/o TNP]	1009.34	1009.91	[M^+]
1358.55	1358.91	[M^+ w/o NP]	1032.90	1032.55	[MNa^+]
1365.58	1365.64	[MK^+ w/o TNP]	1708.37	1708.36	[$2M^+$ w/o 2TNP]
1481.48	1481.31	[MH^+]	1740.44	1740.82	[$2M^+$ w/o 1TNP and w/o 1NP]
1503.46	1503.33	[MNa^+]			
1519.57	1517.99	[MK^+]			
2651.65	2651.69	[$2MH^+$ w/o 2TNP]			
2682.70	2682.87	[$2MH^+$ w/o 2NP]			
2806.80	2806.46	[$2MH^+$ w/o 1TNP]			

The expected m/z values for both peptides were observed and labelled on **Fig. S2**. The full fluorescent peptide has an expected m/z [MH^+] = 1481.48 and was observed at m/z [MH^+] = 1481.31 (**Table S2**). The signal for [M^+ w/o TNP] is observed at m/z = 1326.47, corresponding to the fragmentation by MALDI of the 2-thio-5-nitropyridine (TNP) group (expected m/z = 1326.48). The values for [M+Na^+ w/o TNP], [MK^+ w/o TNP], [MNa^+] and [MK^+] are observed at m/z = 1349.67, m/z = 1365.64, m/z = 1503.33 and m/z = 1517.99 respectively, and correspond to the peptide with or without TNP, cationized with sodium or potassium from the matrix solution. Another fragmentation is the loss of the nitropyridine (NP) [M^+ w/o NP] observed at m/z = 1358.91. Finally, the loss of TNP or NP can result in the

formation of dimers molecules, such as [2MH$^+$ w/o 2TNP], [2MH$^+$ w/o 2NP] and [2MH$^+$ w/o 1TNP] observed at m/z = 2651.69, m/z = 2682.87 and m/z = 2806.46 respectively.

Similar fragmentations were observed for the non-fluorescent peptide (PHP). The full-length peptide was observed at m/z [M$^+$] = 1009.91 (expected m/z [M$^+$] = 1009.34) (Table S1). The signal for [MH$^+$ w/o TNP] is observed at m/z = 855.90. The values for [MNa$^+$] and [MK$^+$] are observed at m/z = 1032.55 and m/z = 1043.48 respectively. The signal resulting from the fragmentation during MALDI analysis of the para-nitro group is visible at m/z = 994.46. The formations of dimers molecules, such as [2M$^+$ w/o 2TNP], [2M$^+$ w/o 1TNP w/o 1NP], are observed at m/z = 1708.36 and m/z = 1740.82 respectively.

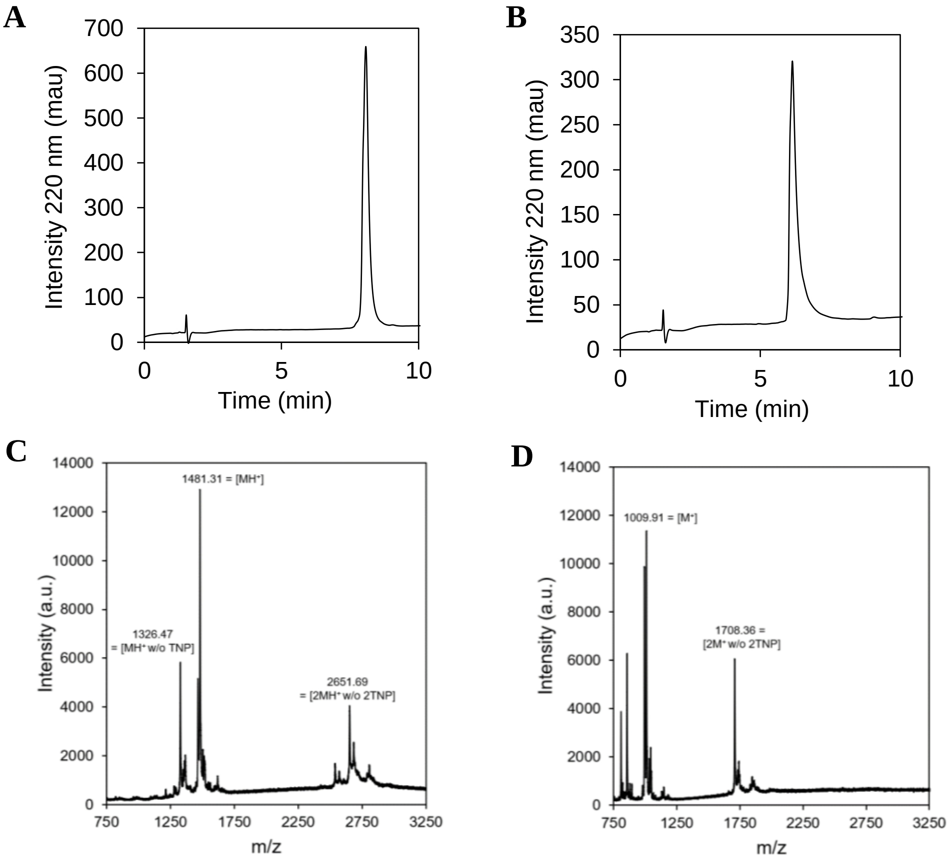

Figure S2. Polyhistidine peptides analysis: (A-B) HPLC analysis of (A) PHP* and (B) PHP (C18, 5-60 % ACN, 10 min). (C-D) MALDI-TOF MS analysis of (C) PHP* and (D) PHP.

PHP grafting onto CS surface:

The DTNP-activated fluorescent peptide was grafted onto fluorescent thiol-coated core-shell NP surface to obtain CS*-PHP* nanoparticles. Conservation of CS stability after this grafting was verified by DLS giving an average diameter of 31.3 nm, while their surface charge, determined by zetametry, was maintained negative at - 41.8 mV (**Table S1**, **Fig. S3.A-C**). Grafting was verified by fluorescence spectroscopy in order to detect both the rhodamine encapsulated in the silica shell of the CS* and the fluorescein of PHP* (**Fig. S3.D-E**).

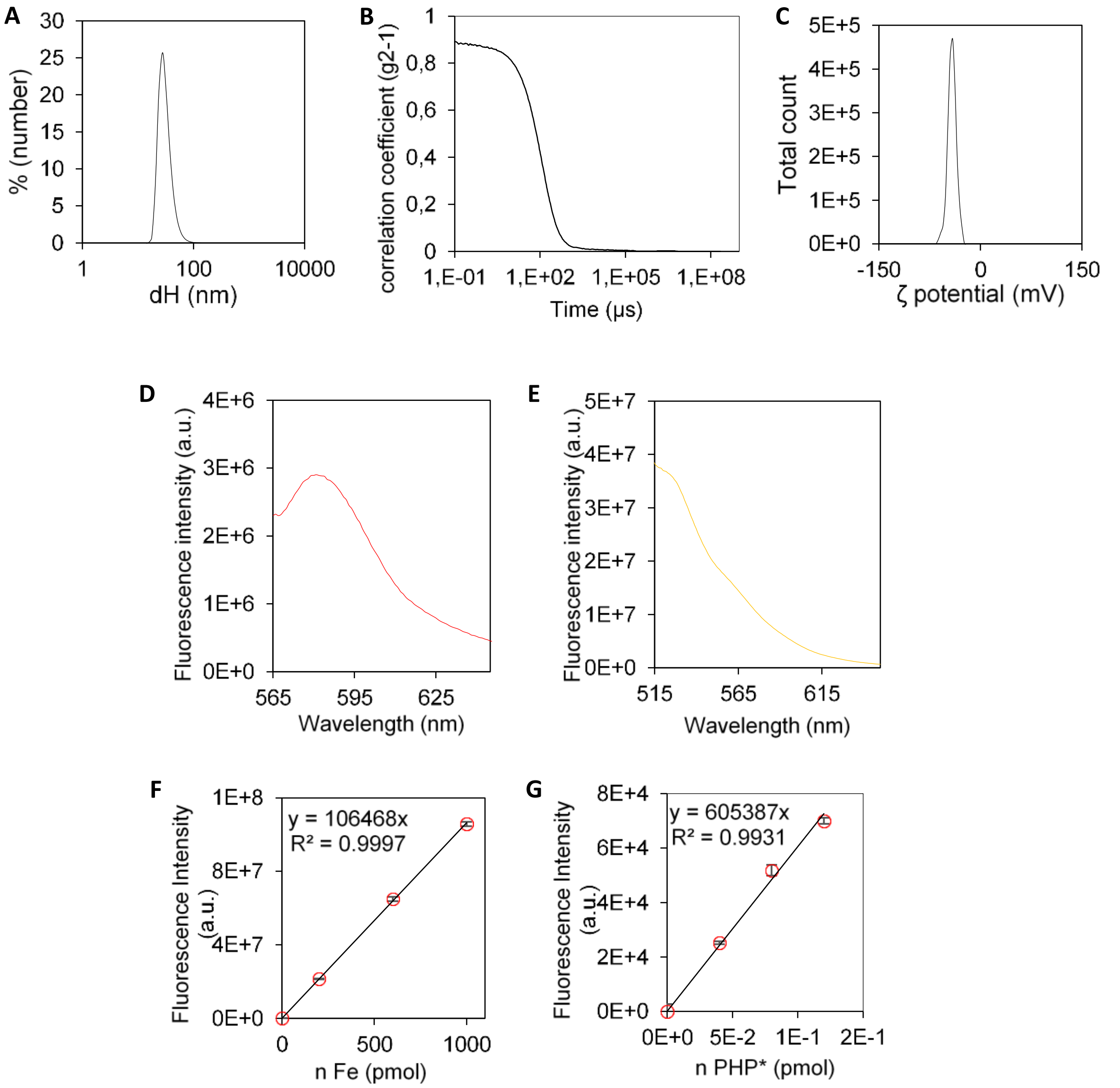

Figure S3. CS*-PHP* nanoparticles characterization: (A) hydrodynamic diameter determined with DLS, (B) DLS correlation function, (C) ζ-potential determined with zetametry. (D-E) Fluorescence spectra of CS*-PHP*: (D) rhodamine from CS* (λ_{em} = 585 nm), (E) CF from

PHP* (λ_{em} = 517 nm). (F-G) Standard curves of PHP* fluorescence intensity (λ_{em} = 517 nm) proportional to (F) iron concentration and (G) PHP* concentration.

- **Optimization of CS*-FAM-PHP synthesis**

To verify CS functionalization, fluorescence intensities of rhodamine inside the silica shell and of FAM were measured (**Fig. S4.D and S4.E**), confirming the grafting of FAM onto the CS* surface. In another experiment, rhodamine-maleimide (ROX) was reacted this time with non-fluorescent CS to form a thioether bond and PHP* (functionalized with fluorescein) linked by a disulphide bond to give CS-ROX-PHP* nanoparticles. This design avoided the overlap of fluorescence which would occur when measuring fluorescence of FAM and PHP*. Fluorescence spectra of ROX and PHP* from CS-ROX-PHP* confirmed the double functionalization of CS (**Fig. S4.F and Fig. S4.G).** This functionalization was also verified in cells and colocalization analysis is presented in the next paragraph. This bifunctionalization strategy was then applied for the synthesis of CS*-FAM-PHP as explained in the main text. The obtained CS*-FAM-PHP and CS-ROX-PHP* were analysed by DLS and zetametry (**Table S1, Fig. S4.A-C**) and their colloidal stability remained intact.

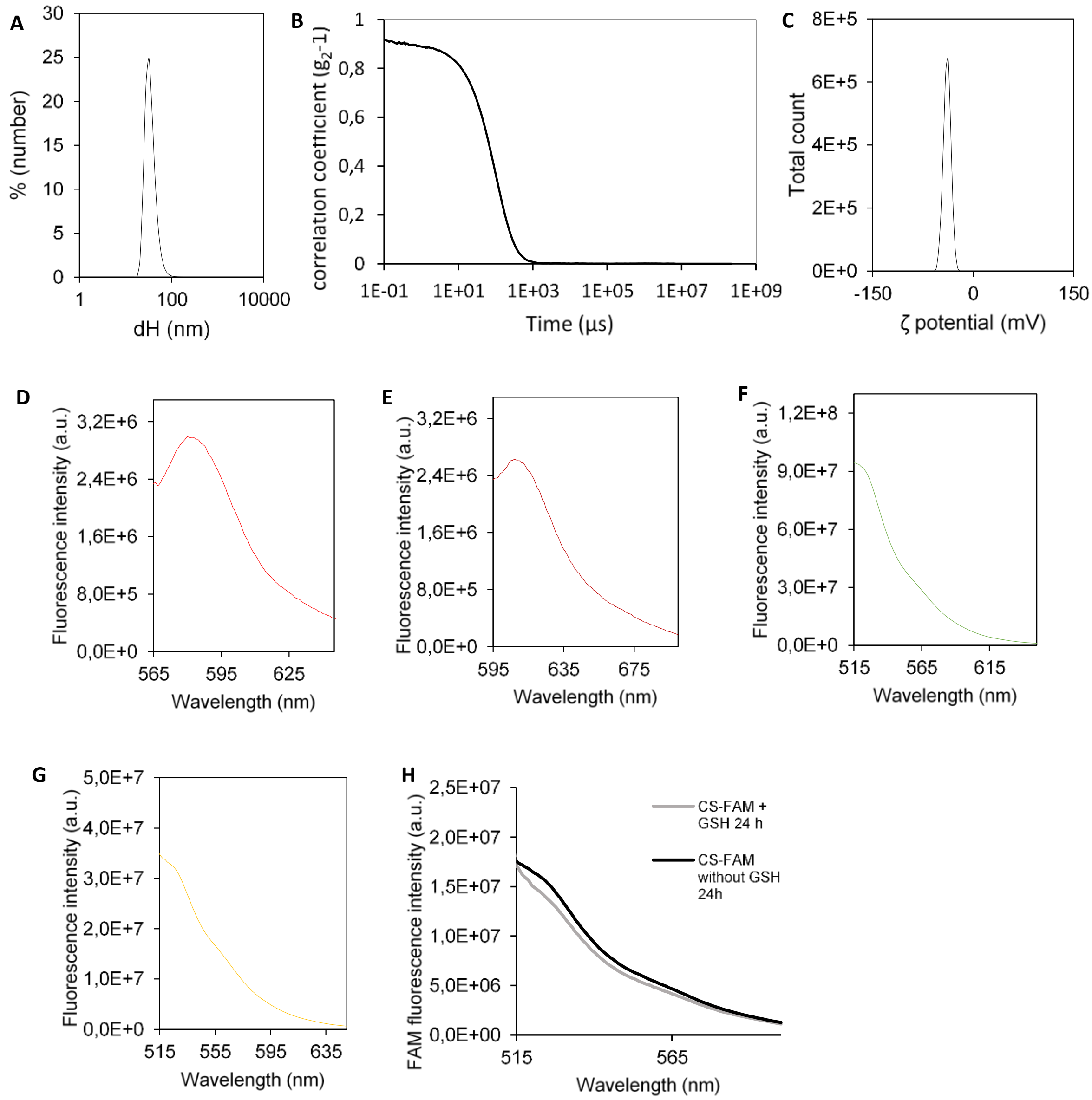

Figure S4. (A-C) Characterization of CS-ROX-PHP* with (A) hydrodynamic diameter determined with DLS, (B) DLS correlation function and (C) ζ-potential determined with zetametry. (D-G) Fluorescence spectra of (D-E) CS*-FAM and (F-G) CS-ROX-PHP*. (D) Rhodamine from the silica shell (λ_{exc} = 540 nm, λ_{em} = 585 nm) and (E) FAM on CS surface (λ_{exc} = 494 nm, λ_{em} = 517 nm). (F) ROX on CS surface (λ_{exc} = 570 nm, λ_{em} = 591 nm) and (G) CF from PHP* (λ_{exc} = 493 nm, λ_{em} = 517 nm). (H) FAM fluorescence intensity spectra of CS-FAM particles incubated or not with glutathione at 10 mM for 24 h.

- **Colocalization analysis: Manders' coefficients calculation and statistical significance**

A control experiment was done by incubating SH-SY5Y cells with CS*-FAM (**Fig. S5**) (FAM being grafted to fluorescent CS* through a thioether stable bond). A colocalization analysis of rhodamine and fluorescein signals (**Fig. S5.A and S5.B** respectively) was performed on these confocal microscopy images in order to confirm FAM covalent grafting onto CS* surface. A simple qualitative evaluation of the fluorescence colocalization of two images is not sufficient to draw conclusions on colocalization and many methods have been developed to fit different situations. Fluorescence observed on merged channels image of CS*-FAM in cells does not appear perfectly yellow as expected for a perfect colocalization of two images (**Fig. S5.C**). Such case is only observed when both images exhibit the same grey level intensity[1]. This may also result from a shift of fluorescence as a consequence of sequential acquisition of images to avoid bleed-through of fluorochromes, as well as difference in fluorescence intensity. To quantitatively evaluate colocalization, the Pearson's coefficient is an intensity correlation coefficient-based analysis that can be determined from a pixel distribution diagram (scatter plot)[2]. From that representation, different types of colocalization can be discriminated. Here, the scatter plot obtained from images presented in **Fig. S5.A-B** clearly displayed a partial colocalization shape-like fluorogram (**Fig. S5.E**) that might result from this shift during acquisition. Also, the fit from this pixel distribution tended to approach the green axe, confirming the difference in fluorescence intensities between both fluorochromes. These results showed that calculation of a Pearson coefficient was not adapted to evaluate colocalization in this case since it considers the average fluorescence intensity pixels values. To prevent this underestimation of colocalization, the Manders' coefficients M1 and M2 were used since they exclude the average intensity values from their equation[3]. These coefficients range from 0 to 1: M1 corresponds to the ratio of red overlapping green and M2 the ratio of green overlapping red. For CS*-FAM observed in cells, the measured Manders' coefficients were M1 = 0.778 and M2 = 0.660, suggesting that both fluorochromes did colocalize. Still, with values near 0.5 such as M2 here, it is intricate to draw accurate conclusions. Costes *et al.* developped a famous algorithm to determine statistical significance of these results: Costes' randomization. Nevertheless, this method relies on the Pearson's coefficient which is not suitable here. Therefore, an object-based and distance analysis developed by Gilles *et al.* was used to determine statistical significance of these colocalization analysis: the centre-to-centre distance between objects from the original image and their nearest neighbour in the other channel are measured and plotted, and the same process is applied to shuffled images (images where objects

from the original image are randomly distributed). The red curve represents the mean distribution of the distances measured on the shuffled images and the curves delimiting the confidence interval appear in green (**Fig. S5.**). The distribution from the original images is represented by the blue curve. The original curve fell outside the confidence interval obtained for random objects locations (green curves). Thus, the colocalization can be considered as statistically significant.[4]. This method has been used here since it doesn't rely on the Pearson's coefficient. These results are gathered in **Fig. S5.F**. Here, the colocalization can be considered as statistically significant, the grafting of FAM onto CS surface was successful and was not altered by cellular internalization. Red fluorescence from rhodamine inside CS* silica shell (**Fig. S5.A**) and green fluorescence from FAM on CS* surface (**Fig. S5.B**) appeared punctual. This experiment showed that CS only functionalized with the fluorophores cannot escape endosomes, even after 24 h incubation. Hereafter, every colocalization analysis performed during this study was conducted using Manders' coefficients along with statistical significance verification.

Double covalent functionalization was also verified by incubating CS-ROX-PHP* with cells for a short time (not long enough to allow endosomal escape). This was essential as we previously showed that species only adsorbed at the surface of CS could get released in cellular environments[5]. Colocalization of ROX and PHP* on CS surface was assessed by confocal microscopy (**Fig. S5.I-K**). Red fluorescence from ROX and green fluorescence from PHP* were still colocalized after 2 h of incubation when NPs were still trapped in endosomes, as shown with measured Manders' coefficients M1 = 0.763 and M2 = 0.804 (**Fig. S5.G**). These results combined with fluorescence spectra (**Fig. S4.E** and **S4.F**) confirmed that the immobilisation of the peptide on CS already functionalized with a fluorescent probe such as ROX was covalent and did not only result from non-specific adsorption of peptides onto NP surface.

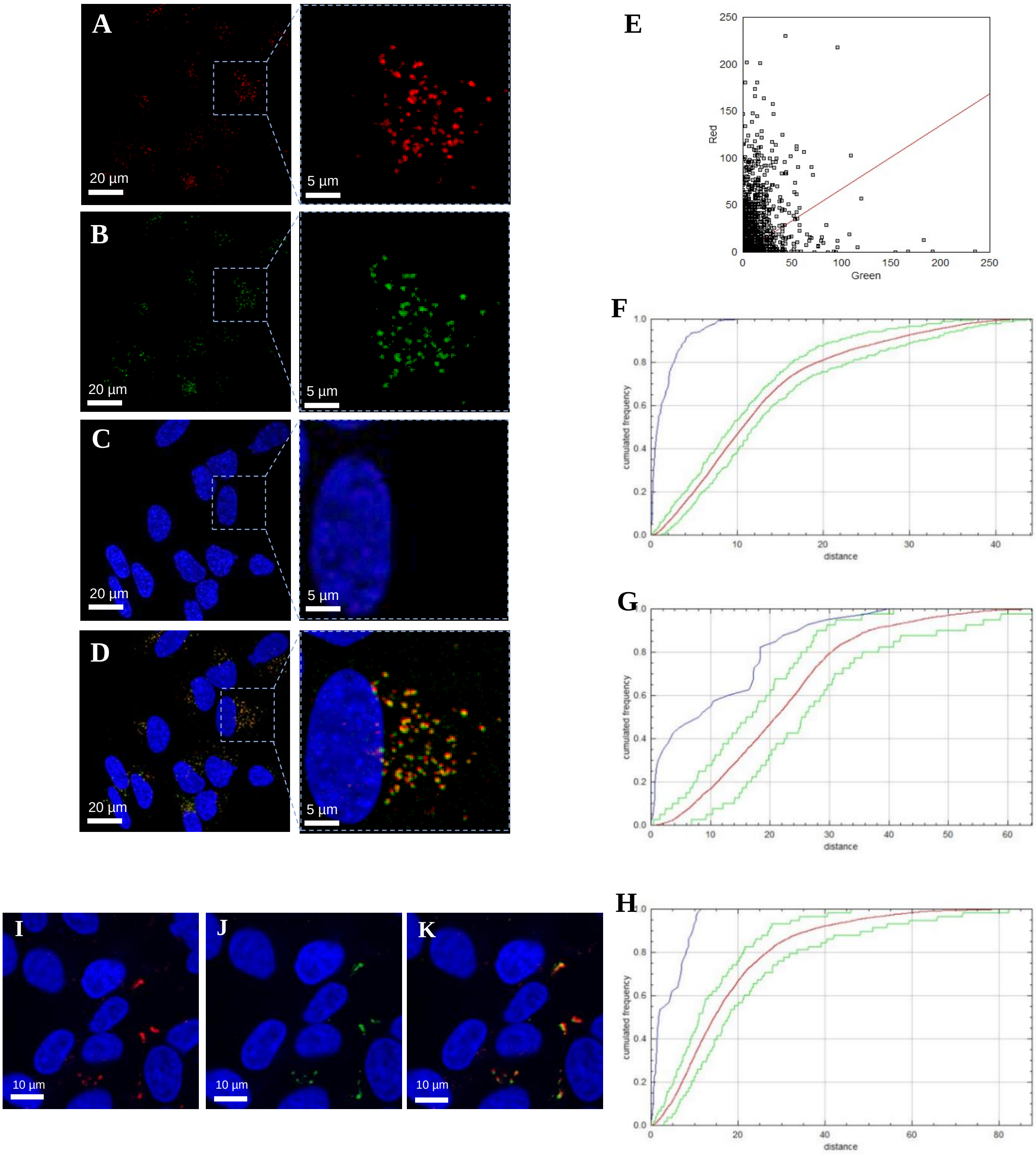

Figure S5. (A-D) Confocal microscopy images on live SH-SY5Y cells incubated with CS*-FAM at [Fe] = 1 mM. Cells were incubated for 4 h with nanoparticles, washed and observed 24 h after incubation. (A) Red fluorescence of rhodamine inside CS*, (B) green fluorescence of FAM on CS* surface, (C) cell nuclei in blue (Hoechst dye 33342) and (D) merged channels. (E) Pearson's scatter plot of CS*-FAM. (F-H): minimum centre-to-centre distance distribution between red and green objects from (F) Fig. S5.A and Fig. S5.B, (G) Fig. S5.I and Fig. S5.J, (H) Fig. 2.C, respectively. Blue curve: distribution for the original images. For green shuffled images (100): the red curve corresponds to the mean distribution and green curves represent the confidence interval. (I-K) Confocal microscopy images on live SH-SY5Y cells, incubated with CS-ROX-PHP* at [Fe] = 1 mM. Cells were incubated for 2 h with nanoparticles. (I) Red

fluorescence from ROX on CS surface, (J) green fluorescence of CF from PHP*, (K) merged channels, with cell nuclei in blue (Hoechst dye 33342).

- **CS stability in DMEM/F12 and cytotoxicity evaluation**

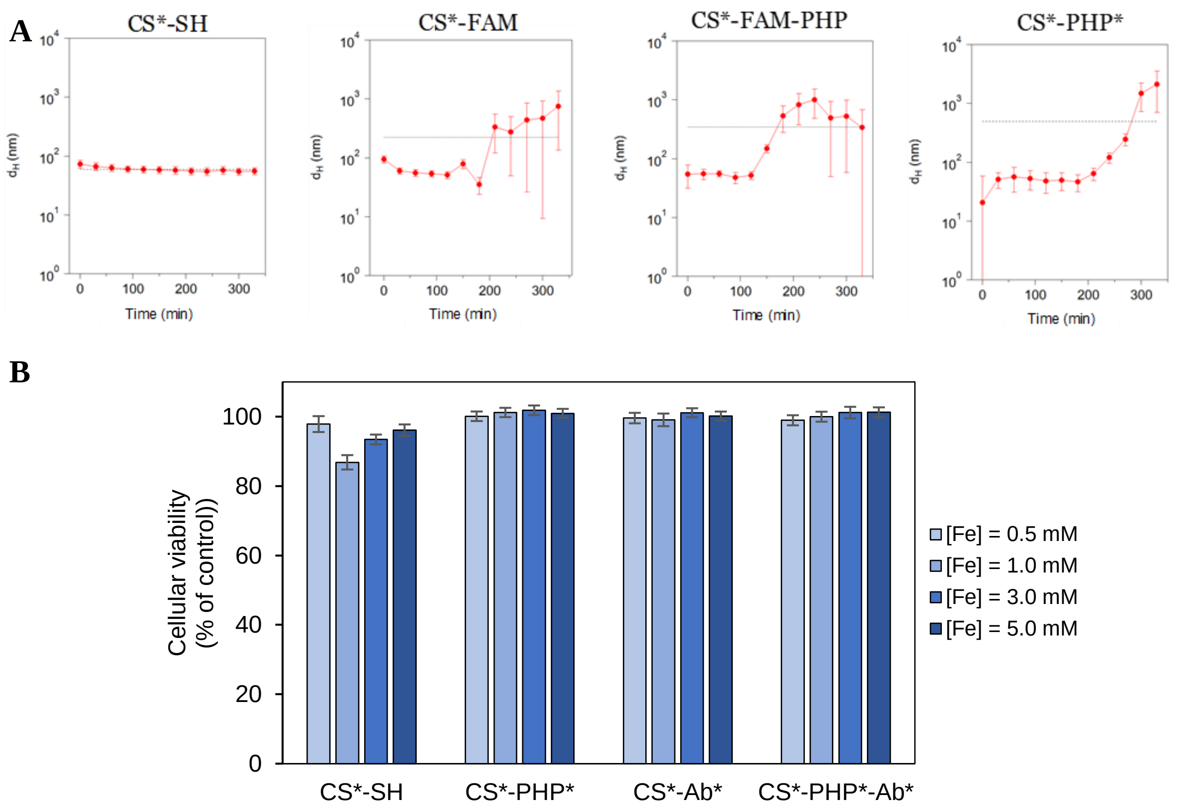

Figure S6. (A) CS stability in cell media at 37 °C determined by DLS (z-average). (B) LDH cytotoxicity assay on SH-SY5Y cells. Cells were incubated with iron concentrations of 0.5, 1.0, 3.0 or 5.0 mM, and cell death was measured 48 h after incubation.

- **<u>Determination of incubation time to observe endosomal escape and necessity for CS surface functionalization with FAM</u>**

SH-SY5Y cells were incubated with CS*-PHP* nanoparticles and observed in confocal microscopy after 2 h, 4 h, 24 h and 48 h (**Fig. S7**). For the 24 h and 48 h conditions, cells were incubated for 4 h with CS, washed and incubated with culture medium without MNPs for 20 h. The green fluorescence of PHP* after 2 h and 4 h was still punctuated and appeared mostly colocalized with rhodamine, suggesting that after these short incubation times, CS*-PHP* nanoparticles were still trapped in endosomes. Eventually, when cells were observed 24 h after incubation, the green fluorescence from the fluorescent peptide appeared diffuse and distributed everywhere in the cell body (**Fig. S7**). In addition, the peptide was no longer colocalized with CS*, suggesting that 24 h was long enough to let particles escape from endosomes and release the peptides from their surface. A slightly more punctual fluorescence was observed after 48 h compared to the 24 h samples that may result from autophagocytosed MNPs. For the subsequent experiments, cells were therefore observed after 24 h of incubation. The diffused fluorescence of PHP* was then easy to detect, in contrast to the red fluorescence from rhodamine inside CS*: red fluorescence became harder to observe when the nanoparticles were spread and diluted in the whole cytosol. Surface functionalization with FAM was therefore necessary to evaluate CS ability to escape endosomes. Moreover, as mentioned in the main text, green fluorescence quenching can be achieved with the use of trypan blue, a membrane-impermeable dye, to ensure the observation of internalized species only. Such quencher molecule is not able to reach fluorophores inside CS silica shell, therefore the grafting of fluorochromes onto CS surface was mandatory.

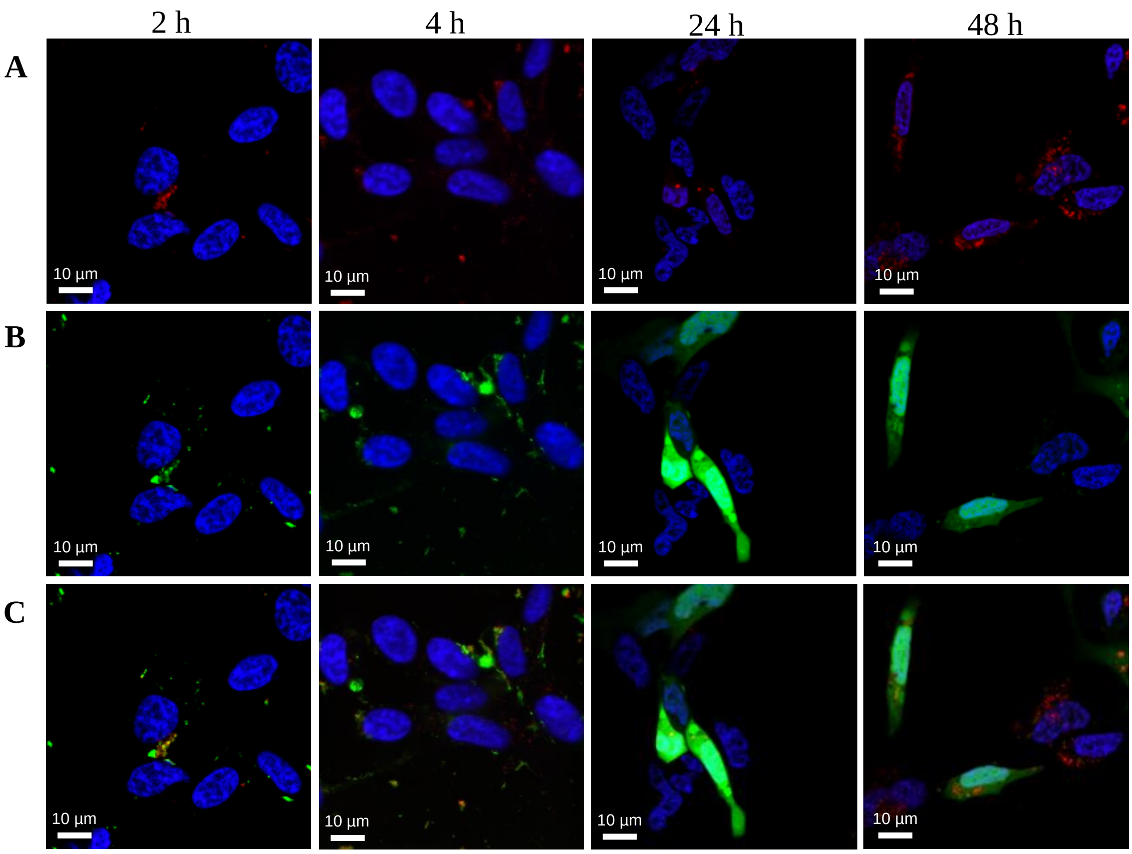

Figure S7. Confocal microscopy images on live SH-SY5Y cells with trypan blue, incubated with CS*-PHP* at [Fe] = 1 mM. Cells were incubated for 4 h with nanoparticles (only 2 h for the 2 h sample), washed and observed 2 h, 4 h, 24 h and 48 h after incubation. (A) Red fluorescence from rhodamine inside CS*, (B) green fluorescence of CF from PHP*, (C) merged channels, with cell nuclei in blue (Hoechst dye 33342).

- **Evaluation of the best parameters to observe cytosolic diffusion of CSs:**

To determine the optimal amount of peptide required for CS efficient release in the cytosol, different amounts of PHP were grafted onto CS surface. The first objects were functionalized with 1250 thiols groups per CS, corresponding to 625 peptides per CS for the CS-FAM-PHP samples. This ratio was later increased since Le Jeune *et al.*[5] used peptide-functionalized CS with 1500 peptides per CS: CS-SH with 1250, 1700, 2100 and 2500 thiol groups per magnetic core were synthesized and their corresponding DLS analysis showed no aggregation despite this increase in functionalization (**Fig. S8.B**). Their surface was functionalized with FAM and PHP, corresponding to 625, 850, 1050 and 1250 peptide molecules per magnetic core and the CS were incubated with cells that were images by confocal microscopy (**Fig. S8**). The diffuse green fluorescence resulting from endosomal escape of CS did not appear stronger when increasing the number of peptides on CS surface. 1250 thiol functions seemed therefore sufficient for further functionalization with 625 peptides per magnetic core.

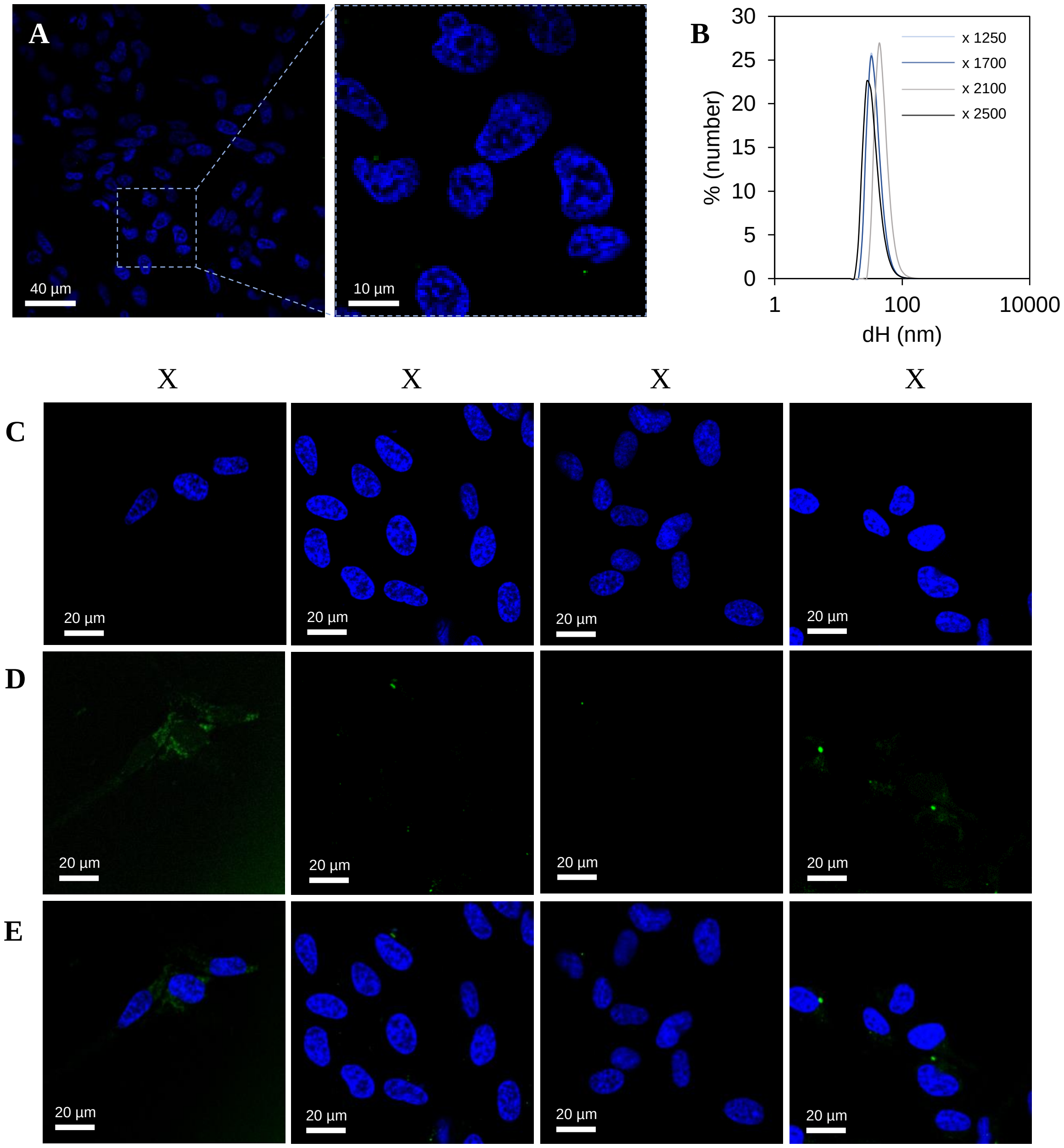

Figure S8. (A) Confocal microscopy images on live SH-SY5Y cells with trypan blue, incubated with PHP* for 4 h, washed and observed 24 h after incubation. Green fluorescence from CF, cell nuclei in blue (Hoechst dye 33342). (B) DLS spectra in number of CS-SH with 1250, 1700, 2100 or 2500 thiol groups per magnetic core. (C-E) Confocal microscopy images on live SH-SY5Y cells with trypan blue, incubated with CS-FAM-PHP at [Fe] = 1 mM with different amounts of peptide functionalization per magnetic core (x 625, x 850, x 1050, x 1250). Cells were incubated for 4 h with nanoparticles, washed and observed 24 h after incubation. (C) Blue fluorescence in cell nuclei from Hoechst dye 33342, (D) green fluorescence from FAM on CS surface and (E) merged channels.

- **Peptide quantification in cells:**

Verification of membrane-bound PHP* complete removal with trypsin/EDTA and TCEP:

In the PHP* quantification experiments by fluorometry, cells were treated after incubation with the nanoparticles with TCEP and trypsin/EDTA in order to eliminate all membrane-bound PHP* peptides, which can include species linked by a disulphide bond to cell surface proteins after thiol/disulphide exchange reactions[6] and species interacting non-covalently with cell-surface components. Control experiments were performed by flow cytometry to check the efficiency of this treatment for membrane-bound species removal. First, we compared for the proteolytic step, digestion with tryspin/EDTA or pronase : two samples of cells incubated with CS-PHP* were treated with TCEP to reduce peptides oxidized at cells' membranes, then either trypsin/EDTA or pronase were added. After washing and centrifugation steps, PHP* fluorescence was measured with flow cytometry (**Fig. S9**). These samples were also tested after adding a trypan blue solution to quench the fluorescence of membrane-bound peptides, trypan blue being non-permeant. No significative difference was observed before and after adding trypan blue, for both conditions (Trypsin/EDTA or pronase). Therefore, it appeared that reduction with TCEP followed by digestion with trypsin/EDTA was sufficient to eliminate all PHP* peptides localized on extracellular membranes. Noteworthy, this allows at the same time for cells to be detached as required.

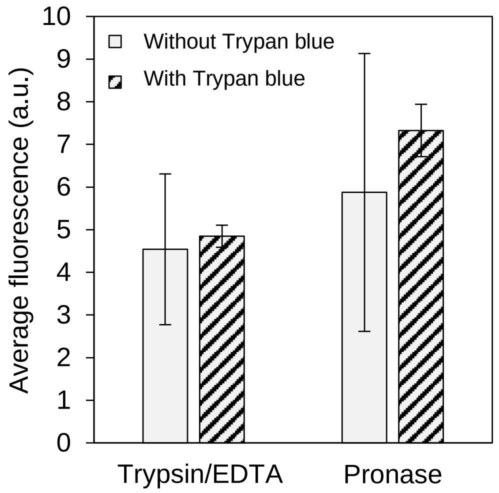

Figure S9. PHP* average fluorescence measured with flow cytometry. Cells were incubated with CS-PHP* ([Fe] = 1 mM) for 24 h and treated with TCEP and Trypsin/EDTA or pronase before measuring PHP* fluorescence. These samples were tested before and after adding trypan blue solution 0.2%.

- **Ab partial reduction:**

For the SDS-Page analysis, when 3 or 5 eq of TCEP were used for reduction, the samples did not give strong bands around 120 kDa, the expected molecular weight for half of the Ab* (**Fig. S10.C**). On the other hand, smaller molecular weight signals, already present in the original Ab* stock solution, appeared more strongly from 10 eq of TCEP, including the wanted one at 120 kDa. Similar signals were visible for 20 eq of TCEP. However, the 80 kDa strip representing a biologically inactive fragment was already pronounced. That is why 10 equivalents of TCEP were selected over 20 to limit the Ab* fraction that lost the antigen recognition site. Higher molecular weight bands corresponded to the full Ab*. This bioactive form could present free thiol groups even without complete separation of both heavy chains and could therefore be suitable for an Ab oriented immobilization. Below 100 kDa, fragments aren't bioactive anymore and were mainly present for TCEP equivalent values greater than 20. In the end, 10 eq of TCEP appeared to be the best amount of reducing agent required for partial Ab* reduction.

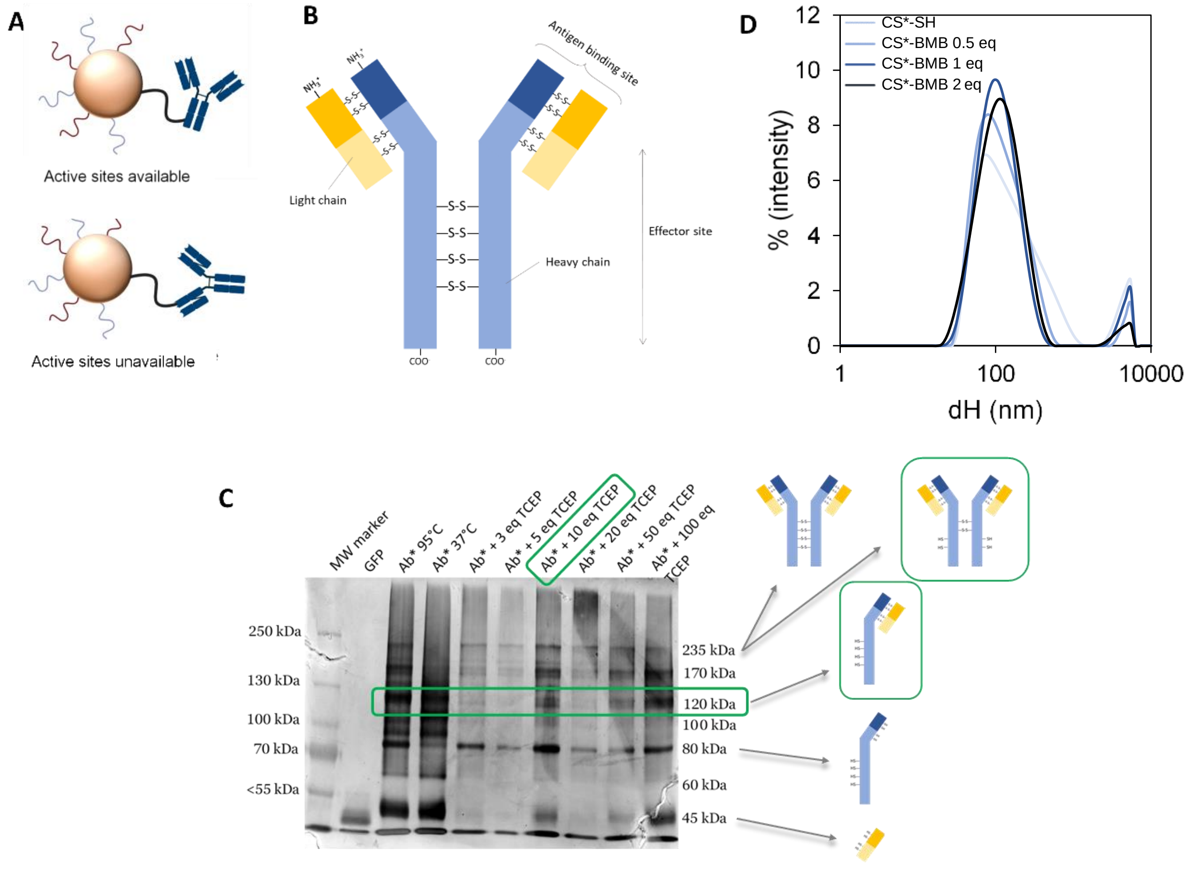

Figure S10. (A) Accessibility differences for antigen binding sites after Ab immobilization onto MNPs' surface and (B) IgG antibody scheme. (C) SDS-Page analysis of Ab* fragments

obtained after reduction with TCEP. (D) Analysis of CS that reacted with different amounts of BMB: hydrodynamic diameter in intensity obtained with DLS.

- **Ab grafting onto CS: optimization without PHP:**

The immobilization of Ab was first optimized without peptide on CS surface (CS*-Ab*). During activation of thiol groups on CS with BMB, there could be an occurrence of cross reactions inter or intra-particles: one maleimide group of the BMB can react with a thiol onto a particle surface, and the other maleimide group of BMB, instead of staying available to later react with the Ab, could react with another thiol group of the same particle or of another particle. Such cross-reaction could lead to particle destabilization and aggregation, especially in the case of reactions inter-particles. To ensure that destabilization of particles did not occur, different amount of BMB were introduced with MNPs. CS*-SH particles, incubated with 0.5, 1 or 2 equivalents of BMB, were then analysed by DLS (**Fig. S10.D**). We observed a slightly downward trend in these values compared to CS*-SH, probably because large particles and aggregated particles were removed in ultrafiltration membranes during washing steps. From these analyses, it was possible to say that BMB grafting onto CS surface, no matter the amount of equivalent of BMB, didn't significantly impact their stability. There is still a risk of thioether bonds formation within one particle. To limit such reaction intra-particle, 2 eq. of BMB per thiol function were introduced.

Fluorescent thiol-coated CS activated with BMB (CS*-BMB) were obtained using two equivalents of BMB *per* thiol function, in order to ensure a complete coverage of thiol groups with BMB and avoid cross-reactions that could lead to aggregation of CS.

For Ab immobilization on CS*-BMB, 1 reduced Ab for 1 CS were first introduced. This grafting was confirmed by fluorescence spectroscopy (**Fig. S11.A**), Ab* being labelled with an allophycocyanin (APC), and its fluorescence was normalized with rhodamine fluorescence from CS*. The extent of potential non-specific adsorption or oxidation of thiols between Ab and CS* was also estimated by mixing CS* and Ab*, without BMB, and found to be negligible (**Fig. S11.B**, "CS*-SH + Ab*"). Two fluorescence emission peaks were detected from APC fluorescence spectrum. The first signal at 665 nm for 1 Ab *per* CS-BMB was stronger than the corresponding control ("CS*-SH + Ab*"), suggesting a successful grafting between Ab* and CS* through BMB. However, these values were quite low since only 1 fluorescent probe was theoretically attached onto CS. To confirm these results, the number of antibody equivalents introduced was increased. The fluorescence intensity detected increased in correlation with the number of Ab* (**Fig. S11.A**), confirming the specific oriented immobilization of Ab* onto CSs

surface. An increase in fluorescence intensity was also observed for control samples, but remained significantly lower than with BMB activation of CS*-SH (**Fig. S11.B**).

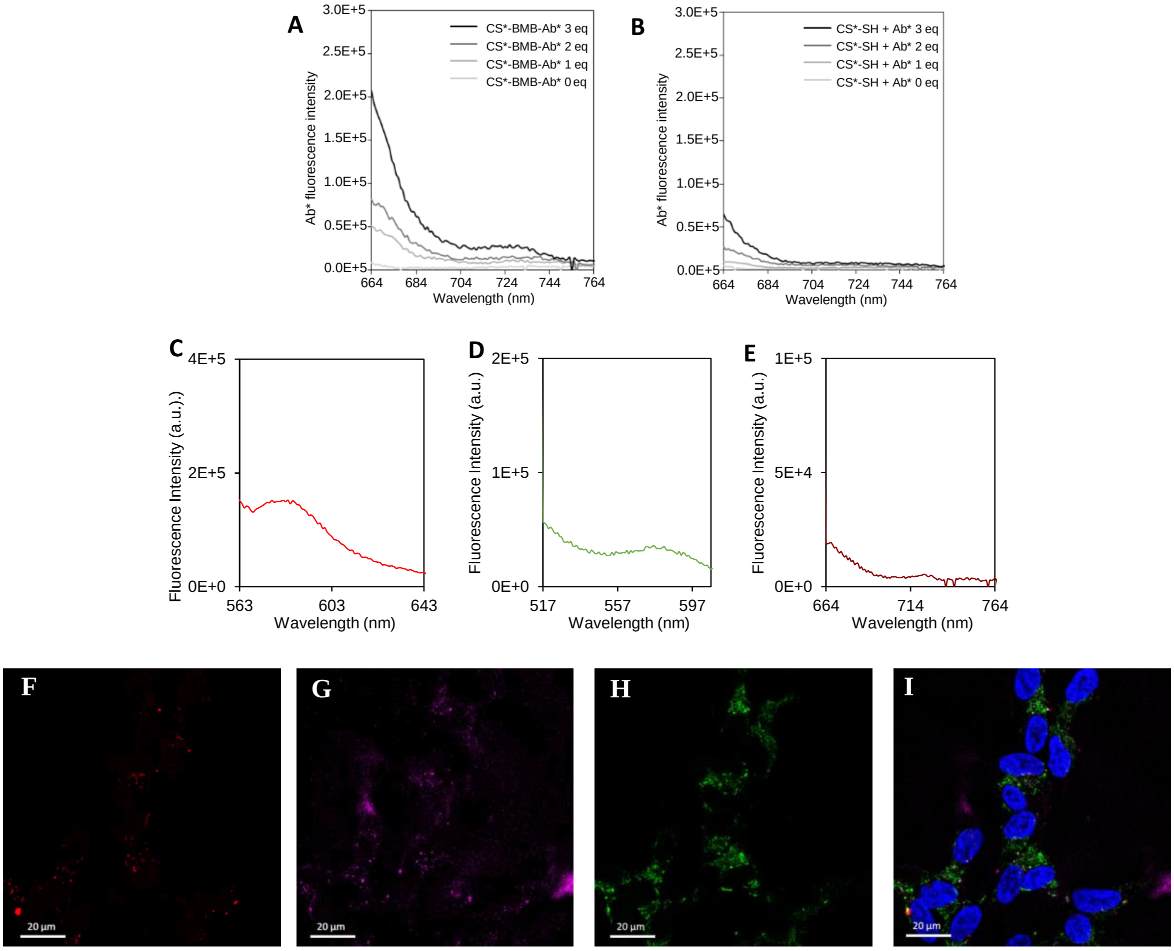

Figure S11. (A-B) Fluorescence spectra of APC from Ab* normalized by rhodamine from CS* (λ_{exc} = 651 nm, λ_{em} = 660 nm) of (A) CS*-BMB-Ab* with different amounts of equivalents of Ab* per CS and (B) CS*-SH with different amounts of equivalents of Ab* per CS to control non-specific adsorption and oxidation of Ab on CSs. (C-E) Fluorescence spectra of CS*-PHP*-Ab*: (C) rhodamine in CS*, (D) CF of PHP* and (E) APC of Ab*. (F-I) Confocal microscopy images on live SH-SY5Y cells incubated with CS*-PHP*-Ab* at [Fe] = 1 mM. Cells were incubated for 4 h with MNPs then washed and observed 24 h after incubation. (F) Red fluorescence from rhodamine inside CS*, (G) APC fluorescence from Ab*, (H) green fluorescence from CF on PHP* and (I) merged channels, cell nuclei in blue (Hoechst dye 33342).

- **Cells transfection to obtain fluorescent HSP27* and colocalization analysis:**

To analyse colocalization between MNPs and HSP27, cells were transfected with a recombinant plasmid to express fluorescent HSP27-GFPs (HSP27*) in the cytosol (**Fig. S12**).

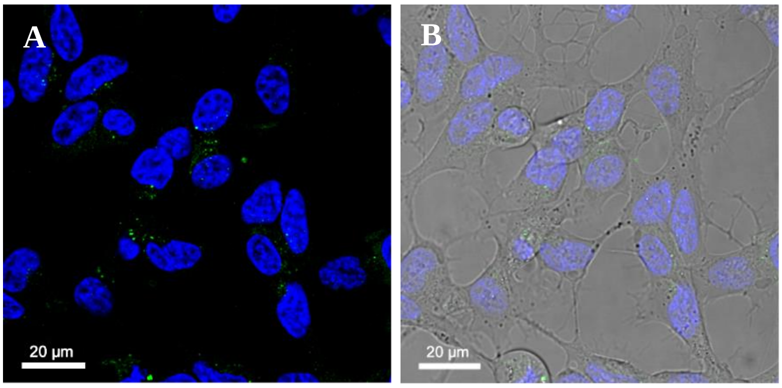

Figure S12. Confocal microscopy images on live SH-SY5Y cells transfected with pEGFP-HSP27. (A) Green fluorescence from HSP27* after transfection and (B) merged channels with green fluorescence and phase contrast. Cell nuclei in blue (Hoechst dye 33342).

- **Targeting capacity evaluation by colocalization analysis in confocal microscopy**

Colocalization of CS* and HSP27* was quantified with Manders' coefficients calculation[1] and compared between different incubation conditions (**Fig 3**, **Table S3**). Coefficients M1 exhibited the ratio of red overlapping green and M2 the ratio of green overlapping red. The use of Manders' coefficients was here the most adapted technique considering the differences between green and red fluorescence detection: on these images, more green fluorescence was observed (HSP27*) than red (CS*), this phenomenon led to M1 coefficients higher than M2 when colocalization occurred (**Table S3**). This time, Manders' coefficients are displayed as percentages and M1 represents the ratio of MNPs that targets HSP27s.

Different iron concentrations for cells incubation were compared: 1 mM or 3 mM. The results presented here were obtained for particles without Ab, that should not be able to target HSP27s. Cells were incubated with a control sample, CS*-SH, without Ab nor PHP, and imaged with confocal microscopy (**Fig. S13.A**). The red fluorescence associated with MNPs appeared punctual and not colocalized with HSP27 green fluorescence according to the corresponding Manders' coefficients (**Table S3**). CS*-SH were not able to target HSP27 because they lacked a targeting agent to do so, but most importantly they remained trapped in endosomes. Similarly, when cells were incubated with MNPs able to escape endosomes, functionalized with the PHP,

but that didn't possess antibodies (CS*-PHP), images didn't show significant colocalization (**Fig. S13.B**, **Table S3**). All the samples that were tested for a 1 mM iron concentration of incubation were incubated again, this time with a three-fold higher concentration (3 mM). As expected, particles that didn't display antibodies at their surface, CS*-SH and CS*-PHP (**Fig. S13.C** and **Fig. S13.D** respectively), maintained similar Manders' coefficients than those obtained for a 1 mM iron concentration of incubation (**Table S3**).

Table S3. Manders' coefficients from confocal microscopy images on cells incubated with different MNPs (functionalized with 0, 1 or 3 Ab / CS), at different iron concentrations (1 mM or 3 mM).

Surface functionalization	Ab/CS	[Fer]	Corresponding image	M1*	M2*	Targeting (+/-)
CS*-SH	0 eq	1 mM	Fig. S13.A	33.5 ± 0.4 %	3.7 ± 4.9 %	-
		3 mM	Fig. S13.C	16.1 ± 2.7 %	6.5 ± 2.7 %	-
**CS*-Ab**	**1 eq**	**1 mM**	Fig. S14.A	**32.3 ± 22.0 %**	18.1 ± 13.7 %	-
		**3 mM**	Fig. S15.A	**83.2 ± 0.7 %**	27.2 ± 1.5 %	++
**CS*-Ab**	**3 eq**	**1 mM**	Fig. S14.C	**46.7 ± 4.0 %**	37.6 ± 5.5 %	+
		**3 mM**	Fig. S15.C	**78.5 ± 11.0 %**	28.5 ± 5.7 %	++
CS*-PHP	0 eq	1 mM	Fig. S13.B	16.4 ± 4.9 %	25.0 ± 12.7 %	-
		3 mM	Fig. S13.D	22.1 ± 18.7 %	12.7 ± 3.2 %	-
**CS*-PHP-Ab**	**1 eq**	**1 mM**	Fig. S14.B	**45.7 ± 10.5 %**	23.4 ± 10.7 %	+
		**3 mM**	Fig. S15.B	**74.9 ± 9.1 %**	25.7 ± 15.1 %	++
**CS*-PHP-Ab**	**3 eq**	**1 mM**	Fig. S14.D	**88.8 ± 4.3 %**	32.1 ± 0.3 %	++
		**3 mM**	Fig. S15.D	**83.6 ± 5.4 %**	31.4 ± 7.2 %	++

* $n = 2$

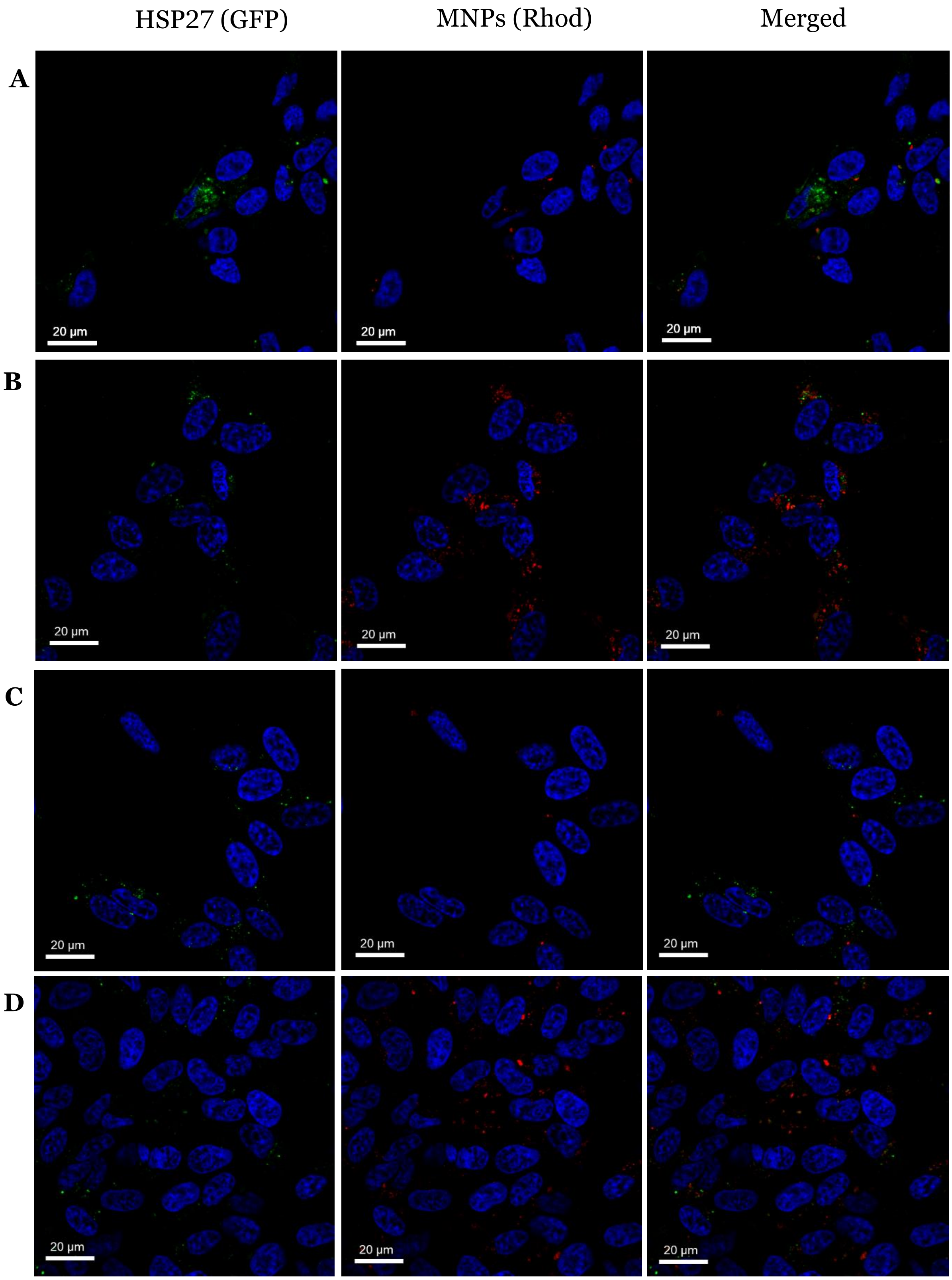

Figure S13. Confocal microscopy images on live SH-SY5Y cells, incubated 4 h with MNPs without Ab and observed 24 h after incubation. (A) and (B) cells incubated with particles at [Fe] = 1 mM and (C) and (D) at [Fe] = 3 mM. (A) and (C) CS*-SH, (B) and (D) CS*-PHP. Cell nuclei in blue (Hoechst dye 33342).

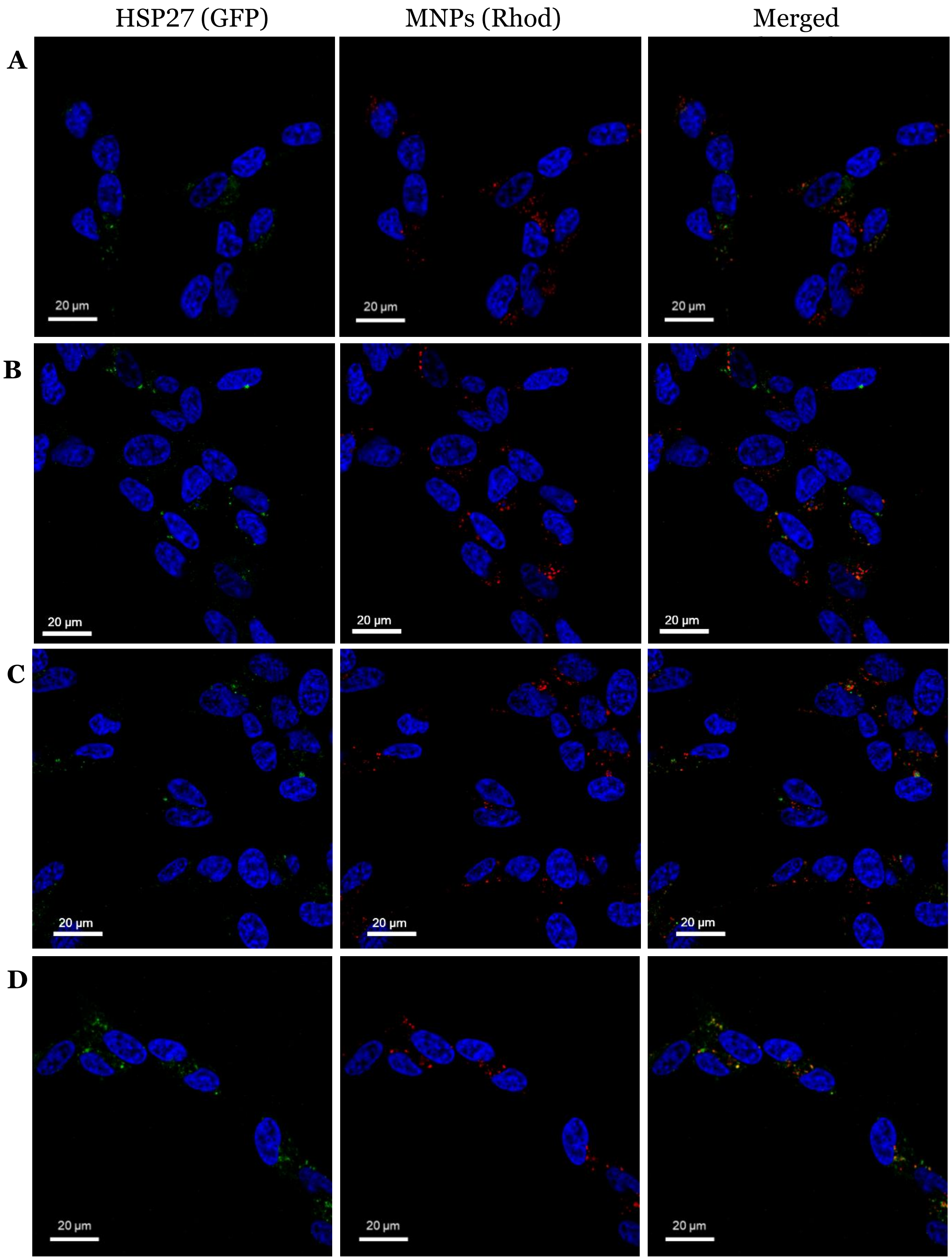

Figure S14. Confocal microscopy images on live SH-SY5Y cells, incubated 4 h with MNPs at [Fe] = 1 mM and observed 24 h after incubation. (A) and (B) CS functionalized with 1 Ab / CS, (C) and (D) with 3 Ab / CS. (A) and (C) CS*-Ab, (B) and (D) CS*-PHP-Ab. Cell nuclei in blue (Hoechst dye 33342).

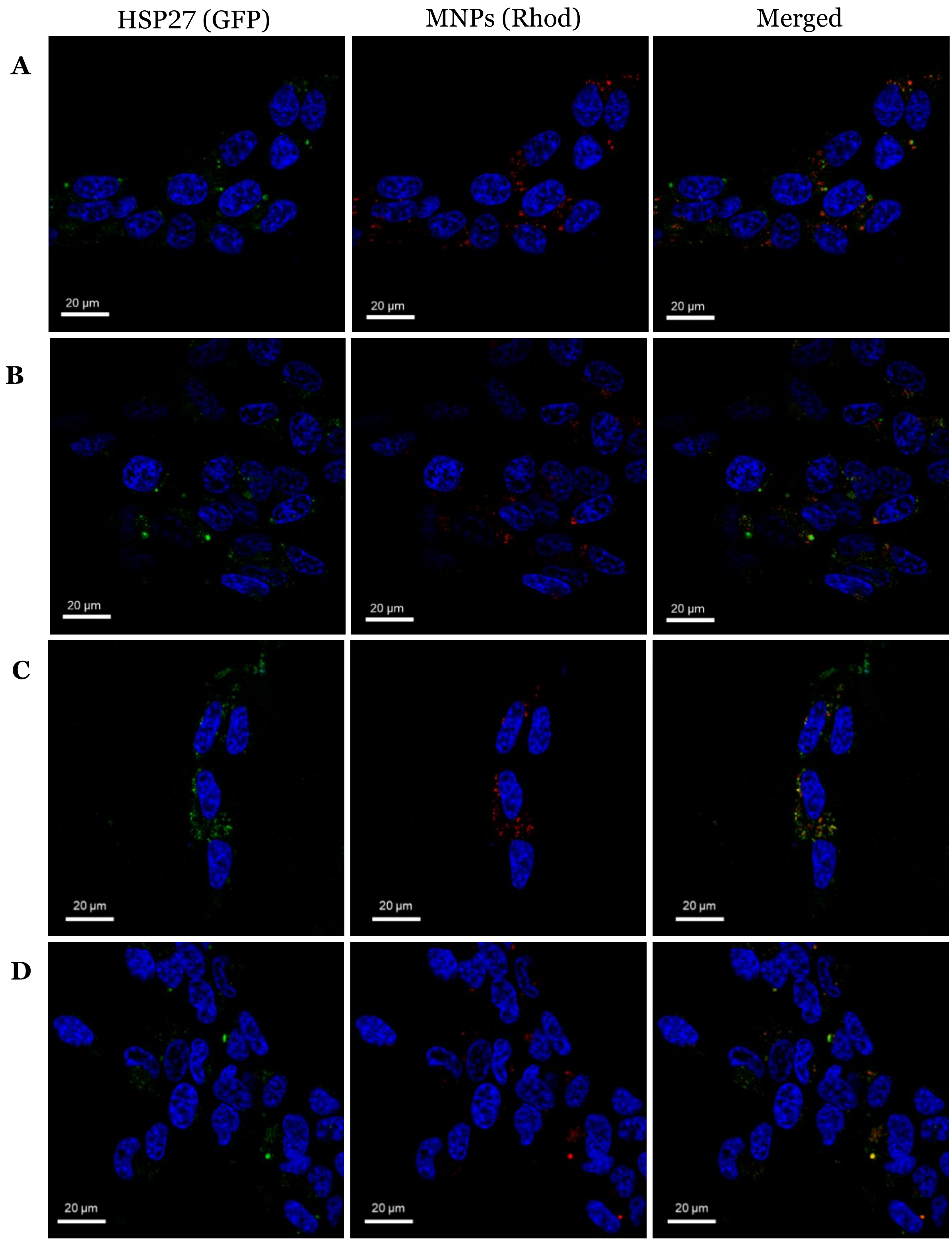

Figure S15. Confocal microscopy images on live SH-SY5Y cells, incubated 4 h with MNPs at [Fe] = 3 mM and observed 24 h after incubation. (A) and (B) CS functionalized with 1 Ab / CS, (C) and (D) with 3 Ab / CS. (A) and (C) CS*-Ab, (B) and (D) CS*-PHP-Ab. Cell nuclei in blue (Hoechst dye 33342).

References :

(1) S. Bolte, F. P. Cordelières *J. Microsc.* **2006**, *224*, 213–232.

(2) K. W. Dunn, M. M. Kamocka, J. H. McDonald *Am. J. Physiol. Cell Physiol.* **2011**, *300* (4), C723-742.

(3) E. M. M. Manders, J. Stap, G. J. Brakenhoff, R. V. Driel, J. A. Aten *J. Cell Sci.* **1992**, *103* (3), 857–862.

(4) J.-F. Gilles, M. Dos Santos, T. Boudier, S. Bolte, N. Heck *Methods* **2017**, *115*, 55–64.

(5) M. Le Jeune, E. Secret, M. Trichet, A. Michel, D. Ravault, F. Illien, J.-M. Siaugue, S. Sagan, F. Burlina, C. Ménager *ACS Appl. Mater. Interfaces* **2022**, *14* (13), 15021–15034.

(6) S. Aubry, F. Burlina, E. Dupont, D. Delaroche, A. Joliot, S. Lavielle, G. Chassaing, S. Sagan *FASEB J. Off. Publ. Fed. Am. Soc. Exp. Biol.* **2009**, *23* (9), 2956–2967.
